# Feline coronavirus drug inhibits the main protease of SARS-CoV-2 and blocks virus replication

**DOI:** 10.1038/s41467-020-18096-2

**Published:** 2020-08-27

**Authors:** Wayne Vuong, Muhammad Bashir Khan, Conrad Fischer, Elena Arutyunova, Tess Lamer, Justin Shields, Holly A. Saffran, Ryan T. McKay, Marco J. van Belkum, Michael A. Joyce, Howard S. Young, D. Lorne Tyrrell, John C. Vederas, M. Joanne Lemieux

**Affiliations:** 1 Department of Chemistry, University of Alberta, Edmonton, T6G 2G2 AB Canada; 2 Department of Biochemistry, Membrane Protein Disease Research Group, University of Alberta, Edmonton, T6G 2R3 AB Canada; 3 Department of Medical Microbiology and Immunology, University of Alberta, Edmonton, T6G 2R3 AB Canada; 4 Li Ka Shing Institute of Virology, University of Alberta, Edmonton, T6G 2E1 AB Canada

**Keywords:** Proteases, Target identification, SARS-CoV-2, X-ray crystallography

## Abstract

The main protease, M^pro^ (or 3CL^pro^) in SARS-CoV-2 is a viable drug target because of its essential role in the cleavage of the virus polypeptide. Feline infectious peritonitis, a fatal coronavirus infection in cats, was successfully treated previously with a prodrug GC376, a dipeptide-based protease inhibitor. Here, we show the prodrug and its parent GC373, are effective inhibitors of the M^pro^ from both SARS-CoV and SARS-CoV-2 with IC_50_ values in the nanomolar range. Crystal structures of SARS-CoV-2 M^pro^ with these inhibitors have a covalent modification of the nucleophilic Cys145. NMR analysis reveals that inhibition proceeds via reversible formation of a hemithioacetal. GC373 and GC376 are potent inhibitors of SARS-CoV-2 replication in cell culture. They are strong drug candidates for the treatment of human coronavirus infections because they have already been successful in animals. The work here lays the framework for their use in human trials for the treatment of COVID-19.

## Abstract (web summary)

Coronavirus main protease is essential for viral polyprotein processing and replication. Here Vuong et al. report efficient inhibition of SARS-CoV-2 replication by the dipeptide-based protease inhibitor GC376 and its parent GC373, which were originally used to treat feline coronavirus infection.

## Introduction

The COVID-19 outbreak evolved into a pandemic due to the virulent nature of SARS-CoV-2, reaching over three million cases worldwide by end of April 2020, with the number of infected growing rapidly worldwide^1^. This current scenario contrasts the more virulent but less efficiently transmitted SARS outbreak in 2002–03, which had only 8000 cases and 774 deaths^2^. There is an urgent need for antiviral therapies for acute COVID-19 infections, especially until an efficacious vaccine is developed.

Coronaviruses are RNA viruses that hijack the host’s translational machinery to generate viral proteins. The viral RNA encodes two overlapping polyproteins: pp1a and pp1ab, which are 450 kD and 750 kD, respectively. The polyproteins need to be cleaved in order to release individual functional proteins for viral replication and transcription. Viral encoded proteases include the main protease (M^pro^), also called 3CL^pro^, and a papain-like protease (PL^pro^). M^pro^ cleaves the polyproteins at 11 positions primarily at conserved Leu Gln | Ser Ala Gly sequences, which allows for assembly of the viral replicase complex^3^.

Given its crucial role in virus replication, the SARS-CoV-2 M^pro^ is a prominent drug target for COVID-19 antiviral therapy. The coronavirus M^pro^ is a cysteine protease for which many different inhibitor classes exist^4^. Protease inhibitors are common drug candidates if they meet the requirements of low toxicity, solubility, and reversibility^5^. Several proteases have been identified as molecular targets and used for the development of novel classes of drugs^5^ including Tipranavir for the treatment of HIV^6^. However, inhibition of cysteine proteases by thiol reactive species is often untenable for human drugs unless the inhibitor is reversible. Michael acceptor drugs that are irreversible in vivo, such as Rupintrivir, have failed in clinical trials owing to low bioavailability^3^. Undesired irreversible reaction occurs with numerous mammalian thiols to destroy the inhibitor. Reaction with host protein thiols could also potentially lead to acute toxicity or immune reaction. In this regard, the reversible reaction of thiols with aldehyde inhibitors to form hemithioacetals presents a unique opportunity for effective cysteine protease inhibition, as they can potentially bind more effectively in the active site of their target protein than with other thiols^7^. Furthermore, water soluble aldehyde bisulphite adducts are readily made reversibly from the parent aldehyde, and revert under physiological conditions^8^. These compounds can be ideal prodrugs for cysteine protease inhibition as described below.

In early studies, we developed peptide-based inhibitors, including aldehydes, against viral cysteine proteases^7^ that were subsequently studied with M^pro^ during the SARS coronavirus (SARS-CoV) outbreak in 2003^9^. Peptide aldehydes and their bisulphite derivatives were later used to inhibit the main protease of the Feline Coronavirus FCoV^10^. FCoV generally causes mild symptoms, but it can lead to feline infectious peritonitis (FIP), which is usually fatal in cats. The bisulphite adduct GC376, a prodrug that converts readily to peptide aldehyde GC373, was well tolerated and able to block the infection in cats^11^. This, along with other studies, that included ferret and mink coronavirus M^pro^, demonstrated the broad specificity of this protease inhibitor^10,12,13^. A crystal structure of GC376 was solved with the homologous Middle East Respiratory Syndrome (MERS) M^pro^ and demonstrated a covalent interaction with the catalytic cysteine of the M^pro^^14^. Recently, structures of the SARS-CoV-2 M^pro^ protease were solved with a peptide-based ketoamide inhibitor^15^ and various re-purposed drugs such as anticancer agents^16^. However, these M^pro^ inhibitors have not been tested in animal models of coronavirus infection nor have they been reported in human or animal trials for SARS. A number of M^pro^ inhibitors are known to have severe side effects and human cell toxicity, especially those that are anticancer agents. In this study, we examine whether GC373 and GC376, established as effective drugs in cats, inhibit SARS-CoV-2 M^pro^ reversibly and have potential for use as antiviral therapy in humans.

In the present study, we examine the use of the prodrug, GC376, and the drug GC373 to test inhibition of the SARS-CoV-2 M^pro^ in vitro. We demonstrate using recombinant SARS-CoV-2 M^pro^ that GC373 and GC376 are potent inhibitors in the nanomolar range. Crystal structures and NMR analysis of SARS-CoV-2 M^pro^ with GC373 and GC376 demonstrates the drug is covalently attached to Cys145 as a hemithioacetal and reveals residues important in inhibitor specificity. In cell culture, we show GC376 and GC373 block virus replication with no toxicity. This study shows efficacy of the feline drug for use against SARS-CoV-2 and a possible treatment for COVID-19.

## Results

### GC373 and GC376 inhibit SARS-CoV-2 M^pro^

**Fig. 1:**
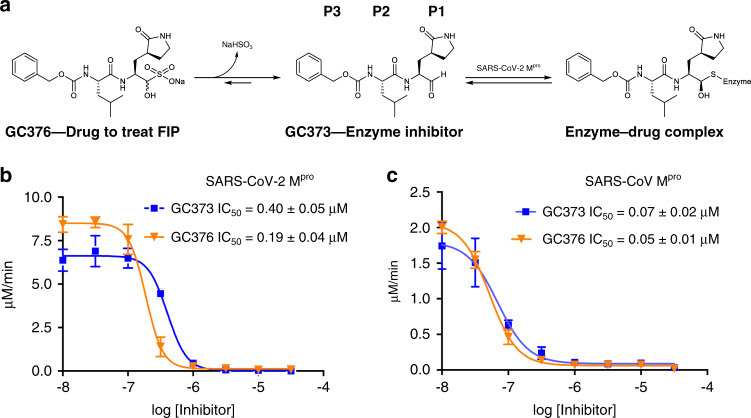
M^pro^ of SARS-CoV-2 and SARS-CoV are inhibited in vitro by GC373 and GC376. **a** Schematic representation of inhibitor prodrug GC376, used to treat cats of FIP, and GC373, the actual protease inhibitor. **b** IC_50_ values for GC373 and GC376 for SARS-CoV-2 M^pro^ and **c** SARS-CoV M^pro^ cleavage of Abz-SVTLQSG-Y(NO2)-R. *N* = 3, values are represented as mean ± SE.

We synthesized the key dipeptidyl compounds, aldehyde GC373 and bisulphite adduct GC376 (Fig. 1a)^10^ (Supplementary Figs. 1–12), to test whether these FCoV and FIPV inhibitors are efficacious toward the M^pro^ of the SARS-CoV-2 and M^pro^ from SARS-CoV, (associated with the 2002 outbreak) which have very similar amino-acid sequences: 95% identity/98% similarity. This compound consists of a glutamine surrogate in the substrate P1 position, a Leu in P2 position, and benzyl group in the P3 position, which reflects the known specificity for the SARS-CoV-2 M^pro^. The SARS-CoV-2 M^pro^ was cloned as a SUMO-tagged fusion protein, which allowed for high-yield expression, enhanced stability, and generation of native N- and C-termini (Supplementary Figs. 13 and 14). Similarly, SARS-CoV M^pro^ was expressed and purified to obtain native N- and C-termini according to previous methods^9^. Kinetic parameters for both the SARS-CoV M^pro^ and SARS-CoV-2 M^pro^ were determined using a synthetic peptide FRET-substrate with an anthranilate–nitrotyrosine donor-acceptor pair (Abz-SVTLQSG-Tyr^NO2^R - Table 1 and Supplementary Fig. 15) as it displays over 10-fold more sensitivity compared with the equivalent EDANS-Dabcyl system^17^. Both SARS-CoV-2 M^pro^ and SARS-CoV M^pro^ exhibited cooperative substrate binding of the FRET-substrate (Supplementary Fig. 16).

**Table 1:** Catalytic parameters of SARS-CoV M^pro^ and SARS-CoV-2 M^pro^.

Protease	K_0.5_ (µM)	k_cat_ (min^−1^)	k_cat_ /K_0.5_ (min^−1^ µM^−1^)	Hill coefficient
SARS-CoV-2 M^pro^	70 ± 10	135 ± 6	1.8 ± 0.4	2.2
SARS-CoV M^pro^	52 ± 17	30 ± 2	0.6 ± 0.2	1.9

Catalytic parameters were determined for SARS-CoV M^pro^ and SARS-CoV-2 M^pro^ with the FRET-peptide substrate Abz-SVTLQSG-Y(NO2)-R. Data are presented as mean values with an *n* = 3 independent experiments. Values are represented as mean ± SEM.

IC_50_ measurements revealed that both GC373 and GC376 inhibit the SARS-CoV M^pro^ and the SARS-CoV-2 M^pro^ in vitro at nanomolar concentrations (Fig. 1b, c). For the SARS-CoV-2 M^pro^, IC_50_ for GC373 and GC376 are 0.40 ± 0.05 μM and 0.19 ± 0.04 μM, respectively. This is in agreement with studies of GC376 with M^pro^ from related viruses. For FCoV M^pro^ the IC_50_ for GC376 was 0.49 ± 0.07 μM, whereas the IC_50_ for GC376 for the M^pro^ of the ferret and mink coronaviruses were 1.33 ± 0.19 μM and 1.44 ± 0.38 μM, respectively^12^. For SARS-CoV M^pro^ we observed an enhanced IC_50_, demonstrating broad inhibition by both compounds, with GC373 and GC376 being 0.070 ± 0.02 μM and 0.05 ± 0.01 μM, respectively. The bisulphite adduct GC376 shows slightly higher potency for both enzymes compared to the free aldehyde. Our in vitro IC_50_ values for GC373 and GC376 reflect tight binding for the SARS-CoV-2 M^pro^ compared with other inhibitors tested in vitro, e.g., ebselen (IC_50_ 0.67 μM)^16^, tideglusib (IC_50_ 1.55 μM)^16^, carmofur (IC_50_ 1.82 μM)^16^, disulfiram (IC_50_ 9.35 μM)^16^, shikonin (IC_50_ 15.75 μM)^16^, PX-12 (IC_50_ 21.39 μM)^16^. Both GC373 and GC376 are also more potent than recently reported ketoamide inhibitors (IC_50_ of the lead compound is 0.67 μM)^15^. Recently, a related peptidyl inhibitor was reported with a similar warhead to our compound, but with an indole group at the P3 position and an IC_50_ of 0.05 ± 0.005 μM^18^. However, that compound has not been demonstrated to be efficacious in animals, as is the case for GC376.

### Crystal structure of SARS-CoV-2 M^pro^ in complex with GC373 and GC376

**Fig. 2:**
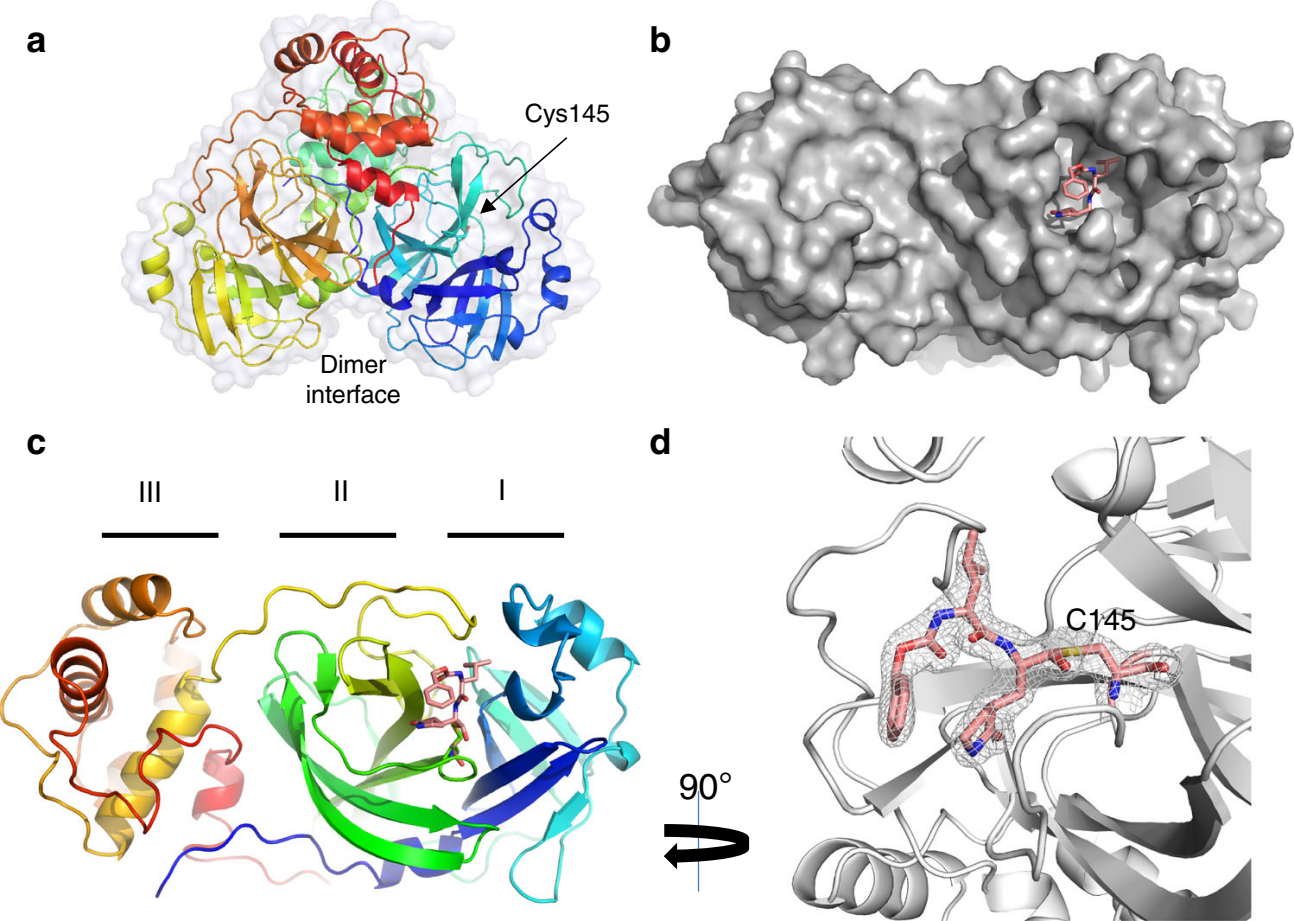
The crystal structure of SARS-CoV-2 M^pro^ in complex with GC373 (converted from GC376). **a** Apo-SARS-CoV-2 M^pro^ forms a dimer (6WTM.pdb). **b** Prodrug GC376, when incubated with SARS-CoV-2 M^pro^, converts to GC373 which forms a covalent bond with C145. Surface representation reveals the active site pocket in complex with GC373 (6WTJ.pdb). **c** Ribbon representation of one SARS-CoV-2 protomer in complex with inhibitor GC373 binding in domain II. **d** GC373 interacts covalently with the active site cysteine of SARS-CoV-2 M^pro^. Electron density at 1σ is shown in gray mesh.

**Fig. 3:**
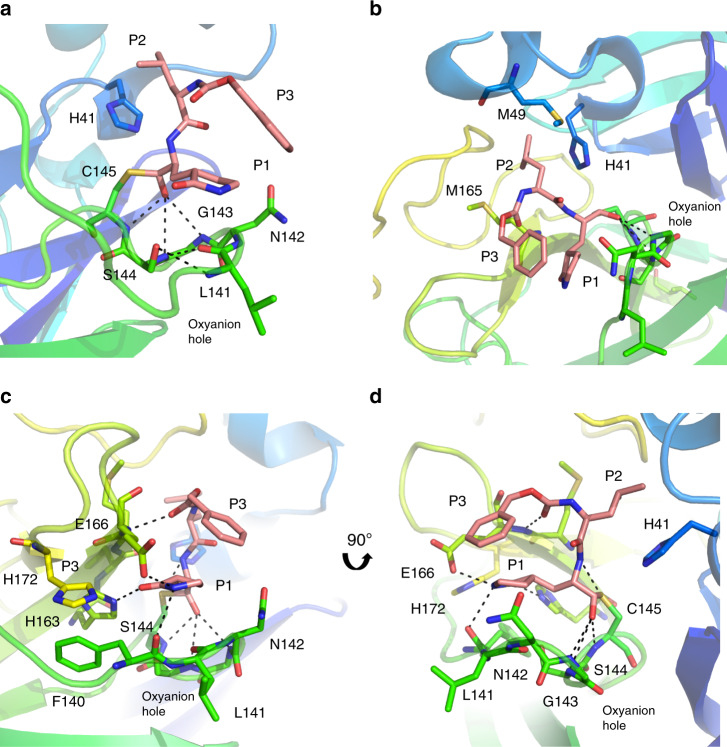
GC373 binds in the active site pocket of SARS-CoV-2 M^pro^. **a** GC373 forms a covalent bond with C145, and the oxyanion is stabilized by backbone H-bonds with G143, S144, and C145. **b** The P2 position of GC373 is stabilized by a hydrophobic pocket by the general base H41, and residues M49, and M165. **c**, **d** H-bonds are established with GC373 and side chains of H163 and E166, as well as backbone of residue E166. SARS-CoV-2 M^pro^ is represented in cartoon representation with the inhibitor in pink. P1, P2, and P3 of the peptidyl inhibitor are indicated. PDB Code:6WTJ.

To gain insight into the mechanism of inhibition, the SARS-CoV-2 M^pro^ crystal structures with inhibitors GC373 and GC376 were determined at 2.0 and 1.9 Å, respectively (Fig. 2 and Supplementary Table 1), and as expected, the prodrug converted to the drug resulting in identical ligands in the structures. The three-dimensional structure of the apo-SARS-CoV-2 M^pro^ (PDB code 6WTM) is highly similar to the recently solved structures with an root-mean-square deviation of 0.19 Å (PDB code 6Y2E)^15,16^. SARS-CoV-2 M^pro^ crystallized as a dimer facilitated by an N-finger of protomer A (residues 1–7) that fits into a pocket in protomer B. Each protomer displayed a two-lobe structure with one lobe composed of two antiparallel β-barrels (Domains I and II), which form a chymotrypsin and 3C-like peptidase fold, with the active site comprised of a Cys145-His41 dyad located at the domain interface. The oxyanion hole, influenced by dimerization^19^, is formed from the main chain residues Gly143, Ser144, and Cys145. The C-terminal domain III is involved in domain swapping and facilitates dimer formation^20^. Molecular replacement with structure 6Y7M.PDB revealed electron density in the Fo–Fc map at the catalytic cysteine for both the prodrug GC376 (PDB Code 6WTJ) and drug GC373 (PDB code 6WTK). In both structures the peptidyl inhibitor is covalently attached to Cys145 as a hemithioacetal, showing that the bisulphite group leaves GC376 (Supplementary Fig. 17). In contrast to the MERS M^pro^-GC376 structure, the SARS-CoV-2 M^pro^ electron density indicated the formation of only one enantiomer for this inhibitor^14^. Backbone contributions from Gly143, Ser144, and Cys145 define the oxyanion hole. The oxyanion hole is further stabilized by a β-turn where the carbonyl of Leu141 hydrogen bonds with the backbone amine of Ser144, and the backbone amide of Leu141 interacts with the hydroxyl of Ser144 (Fig. 3a). Both hydrophobic interactions and a hydrogen bond network is established by residues in Domain II to support inhibitor binding. P2 inserts into a hydrophobic pocket comprised of the general base His41 and residues Met49 and Met165, representing the S2 subsite of the enzyme. (Fig. 3b). The glutamine surrogate in the substrate P1 position forms a hydrogen bond with the side chain of His163 and Glu166 and a hydrophobic interaction with His172. The carbonyl in P3 forms a hydrogen bond with the backbone amide of Glu166 (Fig. 3c, d). Similar to what was observed in the MERS M^pro^-GC376 structure^14^, the benzyl ring and the γ-lactam of the Gln surrogate forms a stacked hydrophobic interaction, which stabilizes the inhibitor in the active site of the protease. Together, this provides strong binding and a low IC_50_ for the inhibitor. A close examination of the subsite for SARS-CoV-2 M^pro^ reveals regions to allow for future inhibitor development (Supplementary Fig. 17).

To confirm the formation of a covalent hemithioacetal as a single enantiomer in the active site, GC373 was prepared with a ^13^C label (>99%) at the aldehyde carbon and mixed in 7.8-fold excess with the M^pro^ protease from SARS-CoV-2 in deuterated buffer. HSQC NMR analysis (700 MHz) showed appearance of a single crosspeak signal (one isomer only) for the hemithioacetal carbon at 76 ppm (^13^C) and 5.65 ppm (^1^H) in accordance with previous chemical shift reports for hemithioacetals^7^ (Supplementary Fig. 18).

### SARS-CoV-2 M^pro^ has enhanced catalytic activity compared to SARS-CoV M^pro^

Recent crystal structural analysis reported differences in the residues residing between the dimer interface of SARS-CoV-2 M^pro^ when compared with the SARS-CoV M^pro^ ^15^. Previous mutagenesis studies, which altered residues at the dimer interface of SARS-CoV M^pro^, and specifically a T285A variant enhanced catalytic activity 3.6-fold^21^. In SARS-CoV-2 T285 is an alanine, and in agreement with this, our analysis shows that the catalytic turnover rate for SARS-CoV-2 M^pro^ (135 ± 6 min^−1^) is almost five times faster than SARS-CoV M^pro^ (30 ± 2 min^−1^) with our substrate Abz-SVTLQSG-Tyr^NO2^R (Table 1). With this FRET substrate, we demonstrate a higher catalytic efficiency with SARS-CoV-2 M^pro^ (1.8 ± 0.4 min^−1^ µM^−1^) compared with SARS-CoV M^pro^ (0.6 ± 0.2 min^−1^ µM^−1^). This finding is in contrast to recent reports where no differences were observed in the catalytic efficiency between SARS-CoV M^pro^ and SARS-CoV-2 M^pro^ using a different substrate: 3011 ± 294 s^−1^ M^−1^ (0.18 ± 0.02 min^−1^ µM^−1^) and 3426 ± 416.9 s^−1^ M^−1^ (0.2 ± 0.03 min^−1^ µM^−1^) respectively^15^. It remains to be determined whether this influences the virulence of SARS-CoV-2.

### GC373 and GC376 are potent inhibitors of SARS-CoV-2 in cell culture

**Fig. 4:**
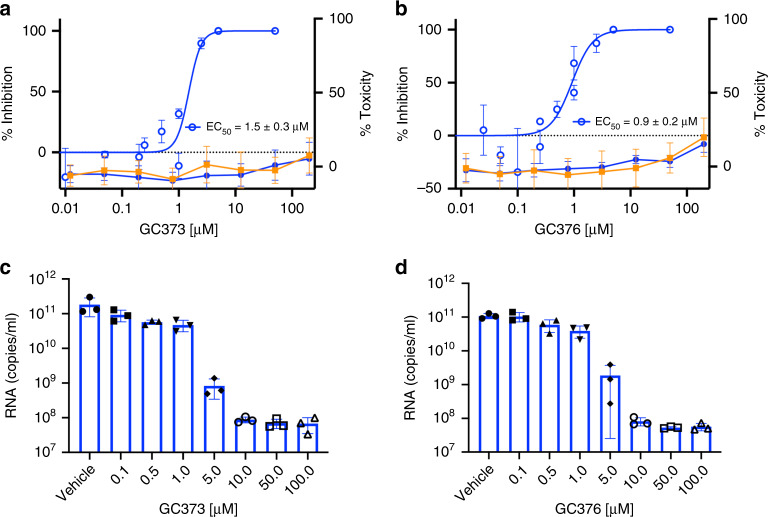
GC373 and GC376 potently inhibit SARS-CoV-2. **a**, **b** SARS-CoV-2 growth in Vero E6 cells was determined by plaque assays 48 h after infection in the presence of various concentrations of drug. Cytotoxicity was measured using the CellTiter-Glo assay. **a** Percent inhibition of SARS-CoV-2 by GC373 in Vero E6 cells (blue open circles) and cytotoxicity in Vero E6 (blue closed circles) and A549 cells (orange). **b** Percent inhibition of SARS-CoV-2 by GC376 in Vero E6 cells (blue open circles) and cytotoxicity in Vero E6 (blue, closed circles) and A549 cells (orange). **c**, **d** To measure the reduction in virus yield, Vero E6 cells were infected with MOI = 0.01 of SARS-CoV-2 in triplicate without or with various concentration of GC373 **c** or GC376 **d** for 24 h and the supernatants harvested, RNA isolated and quantified by qRT-PCR. In the EC_50_ curves, values are represented as mean ± SD of at least two independent experiments. For toxicity assay, values are represented as mean ± SD of 12 independent experiments. RNA data are presented as mean ± SD, with *n* = 3 independent experiments and individual data points shown.

To test the ability of GC373 and GC376 to inhibit SARS-CoV-2, plaque reduction assays were performed on infected Vero E6 cells in the absence or the presence of increasing concentrations of either GC373 (Fig. 4a) or GC376 (Fig. 4b) for 48 h. The results were plotted as a percent inhibition of the number of plaque-forming units per well. The EC_50_ for GC373 was 1.5 μM, whereas the EC_50_ for GC376 was 0.92 μM. To examine cell cytotoxicity, ATP production was measured using the CellTiter-Glo assay on either Vero E6 cells or A549 cells incubated in the presence of the inhibitors for 24 h. The CC_50_ values of both GC373 and GC376 were >200 μM. To further examine the antiviral activity of GC373 and GC376 in an assay with a large dynamic range, a virus yield reduction assay was performed. Quantitative reverse transcription polymerase chain reaction (RT-PCR) was performed on supernatants from cells untreated, and GC373- (Fig. 4c) or GC376- (Fig. 4d) treated cell cultures infected at multiplicity of infection (MOI) = 0.01 for 48 h. It was observed that both GC373 and GC376 are potent inhibitors of SARS-CoV-2, decreasing viral titers by 3-log values, compared with a 2-log value decrease in recently published results using other aldehyde compounds^18^. These results indicate that both GC373 and GC376 are potent inhibitors of SARS-CoV-2 with a therapeutic index of >200.

## Discussion

Numerous drugs were designed originally to inhibit the SARS-CoV M^pro^^3^, however, the SARS outbreak of 2002 was controlled by public health measures and these compounds were never licensed. GC376 has been used to successfully treat FIP in cats as its breakdown product, GC373, effectively inhibits the M^pro^ of FCoV. Analogs of these drugs also inhibited the MERS CoV M^pro^ and blocked viral replication in cells at an EC_50_ of 0.5 μM^14^. Our studies show GC376 and GC373 to be effective inhibitors of SARS-CoV-2 M^pro^, in agreement with previous studies showing GC373 and GC376 possess broad specificity against the M^pro^ of coronaviruses and calciviruses (Supplementary Table 2)^10,13,14,22^. Clearly, these drugs need to be advanced quickly into human trials for COVID-19. SARS-CoV-2 is the cause of COVID-19 and is a virus with a significant mutation rate^23^. Also, in some patients the virus has persisted longer than 2 months with some possibility of re-infection^24^. Vaccines are critically important, but still likely a year or more away as this virus may present vaccine challenges.

There are many clinical trials testing drugs re-purposed from their original indications. Remdesivir, a polymerase inhibitor developed as a treatment for Ebola virus^25^ had initially been used in compassion cases, and is now FDA approved for emergency use in patients with COVID-19^26^. Another drug designed to inhibit RNA-dependent RNA polymerase, including in coronaviruses, is EIDD 2801 (a N4-hydroxycytidine triphosphate that is incorporated into viral RNA to promote errors in progeny RNA). These examples of direct acting antivirals (DAAs) for COVID-19 are critically important^27^. Both GC373 and GC376 are also DAAs designed specifically for coronaviruses. It is likely that several very potent drugs will be required in combination to treat SARS-CoV-2 and to prevent the amplification of drug-resistant mutants^28^, as was the case for HIV^29^. The results of this study show that GC373 and GC376 are candidate antivirals that should be accelerated into clinical trials for COVID-19.

## Methods

### Synthesis of GC373, GC376, and FRET-peptide substrate

Protocols, synthetic details, compound characterization and related methodological information are reported in the Supplementary Methods. Identity and purity (>95%) of all compounds were determined by chromatography (silica or RP-18 HPLC), fully assigned ^1^H- and ^13^C-NMR spectra, and high-resolution mass spectra.

### Cloning, expression, and purification of SARS-CoV M^pro^ and SARS-CoV-2 M^pro^

DNA encoding the main protease M^pro^ from SARS-CoV-2 (Genbank: MN908947.3) was obtained from BioBasic Inc. (Ontario, Canada) and contains an *Eco*RI restriction site upstream and a *Hin*dIII restriction site downstream of the fusion gene. The M^pro^ gene was codon optimized for expression in *Escherichia coli*. The fusion gene was cloned into the *Eco*RI and *Hin*dIII restriction sites of the pET SUMO (small ubiquitin-like modifier) expression vector (Invitrogen) thus ensuring that the SARS-CoV-2 M^pro^ protein is in frame with the His-tagged SUMO protein. The resulting plasmid was transformed into *E. coli* BL21(DE3), induced with 0.5 mM isopropyl β-d-1-thiogalactopyranoside and the protein was expressed for 4–5 h at 37 °C. Cells were harvested by centrifugation (4400 × *g* for 10 min at 4 °C) and suspended in lysis buffer (20 mM Tris-HCl pH 7.8, 150 mM NaCl, 5 mM imidazole). Cells were lysed by sonication, the cellular debris were remove by centrifugation (27,000 × *g* for 10 min at 4 °C), and the supernatant was loaded onto Ni-NTA resin column (Qiagen). After washing the resin with 10 column volumes of lysis buffer containing 20 mM imidazole, the fusion protein was eluted with 40–500 mM imidazole in lysis buffer. Eluted fractions were analyzed by sodium dodecyl sulphate-polyacrylamide gel electrophoresis (SDS-PAGE), the samples with protein of interest were pooled together, dialyzed against lysis buffer containing 1 mM DTT at 4 °C and concentrated using Amicon Ultra-15 filter (Millipore) with a MWCO of 10 kDa. The fusion protein was digested with His-tagged SUMO protease (McLab, South San Francisco, CA) at 4 °C for 1–2 h to remove the SUMO tag. The cleavage mixture was then passed through Ni-NTA resin column. The flow through containing SARS-CoV-2 M^pro^ was collected and analyzed by SDS-PAGE. The SARS-CoV-2 M^pro^ protein was further purified using size exclusion chromatography column (G-100, GE Healthcare,) equilibrated with 20 mM Tris, 20 mM NaCl, 1 mM DTT, pH 7.8. Fractions containing the SARS-CoV-2 M^pro^ protein were pooled and concentrated using Amicon Ultra-15 filter with a MWCO of 10 kDa. In addition, a sample of the fusion protein SUMO-SARS-CoV-2 M^pro^ was also purified using size exclusion chromatography and concentrated as described above. The plasmid encoding the SARS-CoV M^pro^ with an N-terminal His-tag upstream of a FactorX cleavage site was the kind gift of Dr. Michael James. The protein was expressed a purified according to previous protocol^9^.

### Mass Spectrometry of SARS-CoV-2 M^pro^

 The mass of the free SARS-CoV-2 M^pro^ was confirmed by HR-MALDI on a MALDI-TOF (Bruker Ultrafelxtreme, Bruker Daltronics, USA) and LC-MS on an ESI-TOF instrument (Agilent Technologies 6220, CA, USA) using electrospray ionization and analyzed with Agilent Mass Hunter Qualitative Analysis software Package (version B.03.01 SP3).

### Enzyme kinetics of SARS-CoV-2 and SARS-CoV M^pro^

A synthesized fluorescent substrate containing the cleavage site (indicated by the arrow, ↓) of SARS-CoV-2 M^pro^ (Abz-SVTLQ↓SG-Tyr(NO_2_)-R) was used for the fluorescence resonance energy transfer (FRET)-based cleavage assay^15^. The protease reactions of both SARS-CoV-2 M^pro^ and SARS-CoV M^pro^ toward fluorescent substrate were performed in activity buffer (20 mM Bis-Tris, pH 7.8, 1 mM DTT) at 37 °C for 10 min. The final concentration of proteases used in the assay was fixed at 80 nM and the concentrations of the substrate were varied from 0.1 to 500 μM. Reaction was started with the enzyme and the fluorescence signal of the Abz-SVTLQ peptide cleavage product was monitored at an emission wavelength of 420 nm with excitation at 320 nm, using an Flx800 fluorescence spectrophotometer (BioTek). Before kinetic calculations, it was verified that the proportionality between the fluorescence emitted and the amount of the substrate used in the assay was linear. The minimal concentration of the enzyme and time of reaction that gave a linear dependence of amount of generated product with time was chosen. Initial velocities in corresponding relative fluorescence units per unit of time (ΔRFU/s) were converted to the amount of the cleaved substrate per unit of time (μM/s) by fitting to the calibration curve of free Abz-SVTLQ. All data were corrected for inner-filter effects by an adopted literature protocol^17^. In short, the fluorescence signal (RFU) at each substrate concentration was determined and defined as f(FRET). Then, 5 μL of free Aminobenzoyl-SVTLQ at final concentration of 5 μM was added to each substrate concentration and fluorescence was measured: f(FRET + Aminobenzoyl-SVTLQ). Simultaneously, a reference reading was taken with the same free Aminobenzoyl-SVTLQ concentration and was defined as f(ref). The inner-filter correction was obtained as: corr% = (f (FRET + aminobenzoyl-SVTLQ) − f (FRET))/f (ref) × 100%. The corrected initial velocity of the reaction was calculated as *v* = *v*_o_/(corr%). *v*_o_ represents the initial velocity of each reaction.

Kinetic parameters (*V*_max_ and *K*_m_) were derived by fitting the plot of corrected initial velocities versus substrate concentrations to the Michaelis–Menten equation, *v* = *v*_max_ × [*S*]/(*K*_*m*_ + [*S*]) using GraphPad Prism software (GraphPad **8.3.1**). *k*_cat_/*K*_*m*_ was calculated according to the equation, *k*_cat_/*K*_m_ = *v*_max_/([*E*] × *K*_m_). Triplicate experiments were performed for each data point, and the value was presented as mean ± standard deviation (SD).

### Inhibition parameters

Stock solutions of GC373 and GC376 were prepared with 10% aqueous dimethyl sulfoxide (DMSO). For the determination of the IC_50_, 80 mM of SARS-CoV-2 M^pro^ was incubated with GC373 or GC376 at various concentrations from 0 to 100 μM in 20 mM Bis-Tris, pH 7.8, 1 mM DTT at 37 °C for 10 min. The protease reaction was started by addition of 100 μM of the substrate. The GraphPad Prism software (GraphPad 8.3.1) was used for the calculation of the IC_50_ values. Both inhibitors were tested for non-specific binding by performing a reference titration in the absence of DTT showing no influence in the obtained fluorescence readings (data not shown).

### Crystallization

For crystallization, purified SARS-CoV-2 M^pro^ was dialyzed against buffer containing 10 mM NaCl and 5 mM Tris-HCl pH 8.0 overnight at 4 °C, and concentrated with a Millipore centrifugal filter (10 kDa MW cutoff) to 9 mg/mL. Protein was incubated with five molar excess of inhibitor at 4 °C for 2 h prior to crystallization. For SARS-CoV-2 M^pro^, the protein was subjected to the PACT crystallization screen (Molecular Dimensions), with hits identified in several conditions for both inhibitors. For Apo- SARS-CoV-2 M^pro^ the best crystals were observed with hanging drop trays at room temperature at a ratio of 1:1 with mother liquor 0.2 M sodium sulfate, 0.1 M Bis-Tris propane pH 6.5, and 20% w/v PEG 3350. The SARS-CoV-2 M^pro^ with inhibitors crystallized with mother liquid containing 0.2 M sodium chloride 0.1 M 4-(2-hydroxyethyl)-1-piperazineethanesulfonic acid (HEPES) pH 7.0, and 20% w/v PEG 6000. Prior to freezing, crystals were incubated with 15% glycerol as a cryoprotectant. Crystals were initially screened on our 007 MicroMax (Rigaku Inc) home source with crystal screening and final data collection at Stanford Synchrotron Radiation Lightsource (SSRL), beamline 12–2 with Blu-Ice using the Web-Ice interface^30^.

### Diffraction data collection, phase determination, model building, and refinement

All diffraction data sets were collected using synchrotron radiation of wavelength 0.97946 Å at beamline 12–2 of SSRL California, USA, using a Dectris PILATUS 6 M detector. Several data sets were collected from the crystals of SARS-CoV-2 M^pro^ free enzyme as well as with GC376 and GC373 treated. XDS2^31^ and Scala were used for processing the data sets. The diffraction data set of the free SARS-CoV-2 M^pro^ was processed at a resolution of 1.75 Å, in space group P21 (Supplementary Table 1). For the complex of SARS-CoV-2 M^pro^ with GC376 and GC373, the data set collected, was processed at a resolution of 1.9 Å and 2.0 Å and in space group C2 (Supplementary Table 1). All three structures were determined by molecular replacement with the crystal structure of the free enzyme of the SARS-CoV-2 M^pro^ (PDB entry 6Y7M) as the search model, using the Phaser program from Phenix^32^, version v1.18.1-3855). Ligand Fit from Phenix was employed for the fitting of both inhibitors in the density of pre-calculated map from Phenix refinement, using the ligand code K36. Refinement of the three structures was performed with phenix.refine in Phenix software. Statistics of diffraction, data processing and model refinement are given in (Supplementary Table 1). The model was inspected with Ramachandran plots and final models displayed using PyMOL molecular graphics software (Version 1.7.6.5 Schrödinger, LLC).

### Determination of EC_50_ by plaque reduction assay

SARS-CoV-2/CANADA/VIDO 01/2020 was a kind gift from Darryl Falzarano (University of Saskatchewan). Vero (Female green monkey kidney) E6 cells were infected with an MOI of 0.0001 pfu/cell in infection medium consisting of Dulbecco’s Modified Eagle’s medium supplemented with 1× non-essential amino acids (Gibco), 10 mM HEPES, 2% fetal bovine serum, 50 IU/mL penicillin, 50 IU/mL streptomycin different doses of GC373 or GC376. After 1 h, the infecting medium was removed and monolayers were overlaid with growth media (MEM supplemented with 10 mM HEPES) containing 1.2% Avicel RC-591 (DuPont), containing the relevant dose of either GC373 or GC376. After 48 h, cells were fixed in 10% formaldehyde, and stained using 0.5% (w/v) crystal violet. Plaques were counted and data were plotted as % inhibition vs the log_10_ [drug] using Prism (GraphPad). EC_50_ values were determined using a non-linear regression analysis. Experiments were done in triplicate. Error bars indicate standard deviation. To examine the reduction in secretion of viral RNA by Vero E6 cells caused by GC373 and GC376, cells were infected with SARS-CoV-2 MOI = 0.01 pfu/cell in the presence of varying concentrations of GC373 or GC376, for 1 h, the medium removed and replaced with growth media also containing GC373 and GC376 and supernatants harvested 48 h later and quantified by quantitative RT-PCR.

### Quantification of SARS-CoV-2 viral RNA in cell culture supernatants by qRT-PCR

Cell supernatants (140 µL) were collected at various points after infection, and RNA was isolated using the QIAmp Viral RNA Mini kit as per manufacturer’s instructions (Qiagen). Reverse transcription was carried out on 2 µL using Superscript IV Vilo master mix (Invitrogen). Quantitative PCR was carried out using 2 µL of cDNA in TaqMan Fast Master mix using primers and probe for the N gene (N2 primers) designed by the United States center for disease control and prevention (IDT cat#10006606). A standard curve was generated using dilutions of positive control standards from CDC (IDT cat # 10006625).

### Measuring cytotoxicity in A549 and Vero E6 cells

Cell viability was measured using the CellTiter-Glo luminescent cell viability assay (Promega). Either A549 (male human lung epithelial) cells or Vero E6 cells were seeded at 5 × 10^3^ cells/well in 96-well plates and incubated overnight before treatment. Compounds GC373 and GC376 were solubilized in DMSO and added to cells in an eight-point four-fold serial dilution (200 µM to 0.0122 µM). Cells were incubated in the presence of compounds for 24 h before addition of the luminescence substrate and measurement of ATP activity according to manufacturer’s instructions. The percentage of viable cells was calculated relative to cells treated with solvent alone (0.5% DMSO). Results were plotted as the mean of three independent experiments ±SD, where each experiment consisted of quadruplicate wells per concentration of compound.

### Reporting summary

Further information on research design is available in the Nature Research Reporting Summary linked to this article.

## Supplementary information

Supplementary Information File

Reporting Summary

## Data Availability

The authors declare that the data supporting the findings of this study are available within the article, Supplemental Data, and Supplemental Method files. The model and map for the SARS-CoV-2 protease, apo-form, with drug GC373, and with prodrug GC376, are available at the RCSB Protein Data Bank PDB 6WTM, 6WTK and 6WTJ respectively. The NMR data can be accessed here [http://deposit.bmrb.wisc.edu/author_view/BMRB/50262_hy_lssvgcxu.str]. Source data are provided with this paper.

## Source data

Source Data
