# Author Correction: Feline coronavirus drug inhibits the main protease of SARS-CoV-2 and blocks virus replication

**DOI:** 10.1038/s41467-020-19339-y

**Published:** 2020-10-20

**Authors:** Wayne Vuong, Muhammad Bashir Khan, Conrad Fischer, Elena Arutyunova, Tess Lamer, Justin Shields, Holly A. Saffran, Ryan T. McKay, Marco J. van Belkum, Michael A. Joyce, Howard S. Young, D. Lorne Tyrrell, John C. Vederas, M. Joanne Lemieux

**Affiliations:** 1 Department of Chemistry, University of Alberta, Edmonton, T6G 2G2 AB Canada; 2 Department of Biochemistry, Membrane Protein Disease Research Group, University of Alberta, Edmonton, T6G 2R3 AB Canada; 3 Department of Medical Microbiology and Immunology, University of Alberta, Edmonton, T6G 2R3 AB Canada; 4 Li Ka Shing Institute of Virology, University of Alberta, Edmonton, T6G 2E1 AB Canada

**Keywords:** Proteases, Target identification, SARS-CoV-2, X-ray crystallography

Correction to: *Nature Communications* 10.1038/s41467-020-18096-2, published online 27 August 2020

The original version of this Article contained the following error:

In the “Methods” subsection ‘Inhibition parameters’, the volume of SARS-CoV-2Mpro incubated with GC373 or GC376 was incorrectly listed as 80 mM; this has been corrected to 80 nM.

This error has been corrected in both the PDF and HTML versions of the Article.

