# Supplementary material for: Feline coronavirus drug inhibits the main protease of SARS-CoV-2 and blocks virus replication
Source: Nat Commun. 2020 Aug 27;11:4282. doi: 10.1038/s41467-020-18096-2 (PMC7453019; doi:10.1038/s41467-020-18096-2)
Supplement: Supplementary file 1 — Supplementary Information File [file 41467_2020_18096_MOESM1_ESM.zip › 41467_2020_18096_MOESM1_ESM.pdf]

Supplementary Methods

Synthesis of GC376

General Characterization Methods

Nuclear magnetic resonance (NMR) spectra was obtained using an Agilent VNMRS 700 MHz of Agilent/Varian VNMRS 500 MHz spectrometer. For ^{1}H (700 and 500 MHz) spectra, δ values were referenced to $CDCl_3$ (7.26 ppm) or $(CD_3)_2SO$ (2.50 ppm), and for ^{13}C (175 or 125 MHz) spectra, δ values were referenced to $CDCl_3$ (77.16 ppm) or $(CD_3)_2SO$ (39.52 ppm) as the solvents. Infrared spectra (IR) were recorded on a Nicolet Magna 750 or a 20SX FT-IR spectrometer. Cast film refers to the evaporation of a solution on an IR plate. Mass spectra were recorded on a ZabSpec IsoMass VG (high resolution electrospray ionization (ESI)). LC-MS analysis was performed on an Agilent Technologies 6220 orthogonal acceleration TOF instrument equipped with +ve and –ve ion ESI-type ionization, and full-scan MS (high-resolution analysis) with two-point lock mass correction operating mode. The instrument inlet was an Agilent Technologies 1200 SL HPLC system.

Reagents and Solvents

All commercially available reagents and protected amino acids were purchased and used without further purification unless otherwise noted. All the solvents used for reactions were used without further purification unless otherwise noted. Dry solvents refer to solvents freshly distilled over appropriate drying reagents prior to use.

Commercially available ACS grade solvents (>99.0% purity) were used for column chromatography without any further purification.

Reaction progress and fractions from column chromatography were monitored by thin layer chromatography (TLC) using glass plates (5 × 2.5 cm) pre-coated (0.25 mm) with silica gel (normal SiO_2, Merck 60 F254). Visualization of TLC plates was performed by UV fluorescence at 254 nm in addition to staining by $KMnO_4$. Flash chromatography was performed using Merck type 60, 230-400 mesh silica gel at elevated pressures.

Synthetic Scheme for GC376

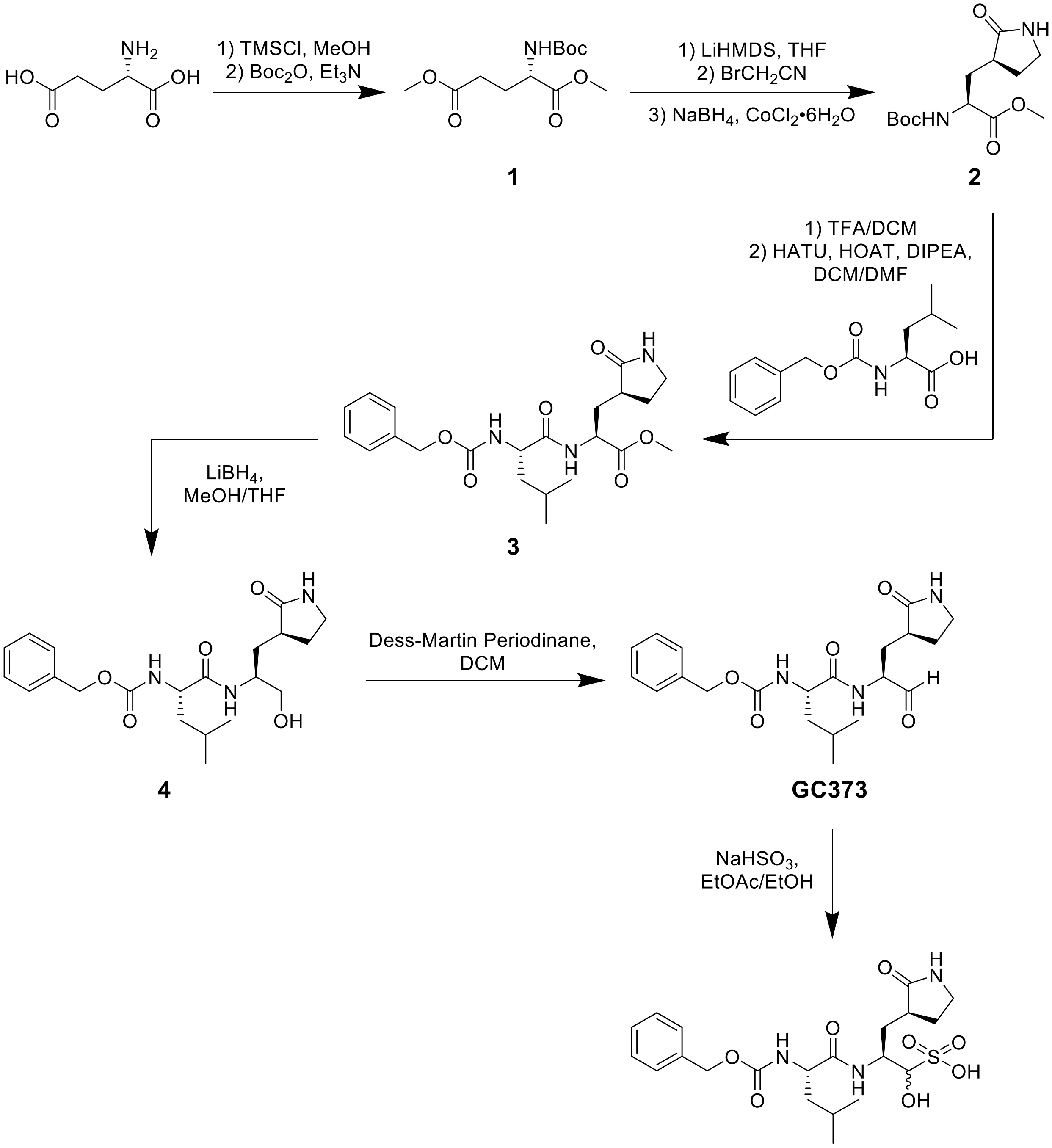

Figure 1. Synthetic scheme for the synthesis of GC376.

Dimethyl (*tert*-butoxycarbonyl)-*L*-glutamate (**1**)

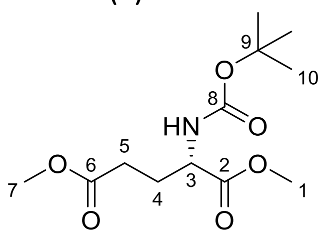

This known compound was synthesized based on a literature procedure[1]. *L*-Glutamic acid (30.00 g, 202.5 mmol, 1.0 equiv) was added to a flame-dried RBF under Ar, followed by the addition of dry MeOH (506.0 mL). The stirred suspension was then cooled to 0 ºC, and TMSCl (113.1 mL, 891.7 mmol, 4.4 equiv) was added slowly over 15 minutes. The reaction mixture was stirred at 0 °C for 1.5 h, and then stirred overnight at rt. Triethylamine (182.5 mL, 1316 mmol, 6.5 equiv) was added to the reaction mixture slowly over 15 minutes. Next, Boc_2O (48.62 g, 222.3 mmol, 1.1 equiv) was added in one portion, and then the reaction mixture was capped with pressure-equalizing Ar and stirred at rt for 3 h. The reaction mixture was then concentrated under reduced pressure to produce a white solid. The solid was triturated by suspension in Et_2O (500 mL), and collected by filtration. The solid was washed with additional volumes of Et_2O (3 × 250 mL), and then concentrated *in vacuo* to produce a crude oil. The crude product was purified using silica column chromatography (50/50 EtOAc/ Hexanes), and yielded a clear, yellow oil (50.44 g, 183.2 mmol, 91%): R_f = 0.61 on SiO_2, 50% EtOAc in hexanes;

IR (DCM cast film, v_{max} / cm^{-1}) 3369, 2979, 2956, 1742, 1717, 1518, 1439, 1392, 1368, 1252, 1212, 1167

^{1}H NMR (500 MHz, $CDCl_3$) δ_H 5.13 (1H, d, *J* = 6.8 Hz, NH), 4.30 (1H, d, *J* = 5.0 Hz, H3) 3.71 (3H, s, H1), 3.65 (3H, s, H7), 2.31 (2H, m, H5), 2.15 (1H, app td, *J* = 13.2, 7.3, H4), 1.92 (1H, app dtd, *J* = 14.6, 8.3, 6.5, H4), 1.40 (9H, s, H10)

^{13}C NMR (125 MHz, $CDCl_3$) δ_C 173.3 (C6), 172.6 (C2), 155.3 (C8), 80.0 (C9), 52.8 (C3), 52.4 (C1), 51.7 (C7), 30.0 (C5), 28.3 (C10), 27.8 (C4)

OR: $[\alpha]_D^{26}$ = 7.20 (*c* = 1.35, DCM)

HRMS: (ESI) Calcd for $C_{12}H_{21}NNaO_6$ $[M + Na]^+$ 298.1261, found 298.1260

(2S)-2-(tert-butoxycarbonylamino)-3-[(3'S)-2'-oxo-3'-pyrrolidinyl]propanoic acid methyl ester **(2)**

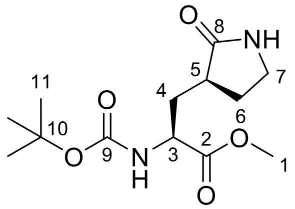

This known compound was synthesized based on a modified literature procedure[1]. **(1)** (1.00 g, 3.62 mmol, 1.00 equiv) was deposited in a flame-dried RBF under argon, to which 10.34 mL of freshly distilled THF was added. The oily starting material was dissolved at rt before LiHMDS (1.0 M in THF, 7.82 mL, 7.82 mmol, 2.16 equiv) was added over a period of 2 min at –78°C. The reaction mixture went from clear and nearly colourless to light yellow, and was allowed to stir at –78 °C for 1 h. Next $BrCH_2CN$ (0.270 mL, 3.88 mmol, 1.07 equiv) was slowly added over a period of 1 h (addition rate of ca. 0.02 mL / 5 min), while maintaining reaction temperature at –78 °C. The reaction mixture was then stirred at –78 °C over a period of 2 h. After 2 h, some starting material remained but the reaction mixture was quenched by addition of 5.2 mL of HCl at –78 °C.

The reaction mixture was then removed from the cooling bath to allow the ice in the flask to melt, stirring for 50 min. The mixture was then extracted with EtOAc (3x). The combined EtOAc layers were washed with H_2O (2x) and brine (1x). Drying over $MgSO_4$ and concentration by rotovap furnishes a dark brown oil. This oil was then dissolved in 5.0 mL of DCM, to which activated charcoal (0.35 g) and silica (1.40 g) was added. The slurry was spun on a rotovap without heat or vacuum for 1 h. Afterwards, the charcoal and silica was filtered through a celite pad and the filtride washed with additional volumes of DCM. Concentration of the filtrate furnishes a yellow oil. This oil was then transferred to a flame-dried RBF under argon, to which $CoCl_2{\bullet}6H_2O$ (0.290 g, 1.22 mmol, 0.67 equiv) was added. The material was then dissolved in 20.0 mL of freshly distilled MeOH. The solution was then cooled to 0 °C and $NaBH_4$ (0.462 g, 12.20 mmol, 6.74 equiv) was added in multiple portions over a period of 30 min. Upon addition of $NaBH_4$, the reaction mixture immediately turned black and started bubbling. Once addition was finished and bubbling slowed, the reaction mixture was capped under a blanket of pressure-equalizing argon, removed from the ice bath, and allowed to warm to rt, stirring for 24 h. After 24 h, the reaction mixture was concentrated on the rotovap to a minimal volume. To the obtained residue was then added 1 M of citric acid at 0 °C. The mixture was further diluted with EtOAc and extracted with EtOAc (3x). The combined EtOAc layers were then washed with sat. $NaHCO_3$ (2x) and brine (1x). Drying over Na_2SO_4 and removal of solvent furnishes a very pale yellow oil as crude. This material was then purified via flash column chromatography using an eluent system of EtOAc. Elution of product was monitored by $KMnO_4$ staining (Rf = 0.16, EtOAc).

Concentration of product fractions and co-evaporation with Et_2O furnishes the desired product as a white solid (0.280 g, 0.973 mmol, 54%).

IR (DCM cast film, v_{max} / cm^{-1}) 3290, 2977, 1745, 1698, 1523, 1440, 1392, 1367, 1276, 1213, 1168

^{1}H NMR (500 MHz, $CDCl_3$) δ_H 6.62 (1H, s, NH), 5.55 (1H, d, *J* = 7.8 Hz, NH), 4.29 (1H, m, H3), 3.72 (3H, s, H1), 3.36 – 3.31 (2H, m, H7), 2.48 – 2.40 (2H, m, H5, H6), 2.11 (1H, ddd, *J* = 14.3, 10.8, 3.7 Hz, H4), 1.88 – 1.40 (2H, m, H4, H6), 1.41 (9H, s, H11)

^{13}C NMR (125 MHz, $CDCl_3$) δ_C 179.8 (C8), 172.9 (C2), 155.8 (C9), 79.9 (C10), 52.4 (C1), 52.3 (C3), 40.4 (C7), 38.2 (C5), 34.1 (C4), 28.3 (C11), 28.1 (C6)

OR: $[\alpha]_D^{26}$ = 3.65 (*c* = 0.57, DCM)

HRMS: (ESI) Calcd for $C_{13}H_{22}N_2NaO_5$ $[M + Na]^+$ 309.1421, found 309.1423

Methyl (*S*)-2-((*S*)-2-(((benzyloxy)carbonyl)amino)-4-methylpentanamido)-3-((*S*)-2-oxopyrrolidin-3-yl)propanoate **(3)**

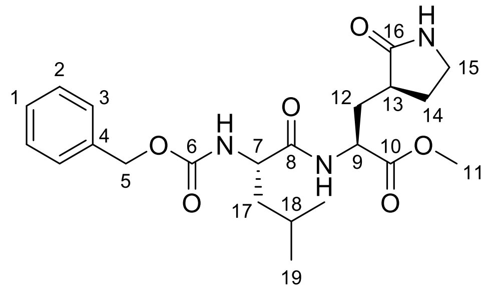

This known compound was synthesized *via* an alternative procedure. **(2)** (4.00 g, 13.97 mmol, 1.0 equiv) was dissolved in 140 mL of 50/50 TFA/DCM and stirred for 1 h at rt, with gas evolution being observed. The solution was then concentrated on rotovap and

co-evaporated with DCM (5x). In a separate flame-dried RBF under argon was deposited Cbz-Leu-OH (90% purity, 4.12 g, 13.97 mmol, 1.0 equiv), HATU (5.31 g, 13.97 mmol, 1.0 equiv). The material was then dissolved in 70 mL of DMF. Next, HOAT (0.6 M in DMF, 2.33 mL, 1.40 mmol, 0.1 equiv) was added, followed by DIPEA (7.30 mL, 41.91 mmol, 3.0 equiv). The reaction mixture turned bright yellow and was incubated for 5 min. The previously concentrated Boc-deprotected material was then dissolved in 70 mL of DCM and added dropwise to the incubating solution, with the bright yellow colour quickly fading. The reaction mixture was the capped under a blanket of argon and allowed to react at rt for 1.5 h. Next, the reaction mixture was diluted with H_2O and EtOAc. The layers were separated and the H_2O layer was further extracted with EtOAc (3x). The combined EtOAc layers were then washed with sat. $NaHCO_3$ (2x), 1 M HCl (2x), and brine (1x). Drying over Na_2SO_4 and concentrating on the rotovap furnishes a crude yellow oil. This material was used without further purification but may be purified by flash column chromatography over silica, using an eluent system of pure EtOAc. Product elution was monitored by TLC and $KMnO_4$ staining (R_f = 0.43, 5/95 MeOH/EtOAc), and concentration of product fractions furnishes a transparent, slightly yellow oil as the desired product (6.056 g, 13.97 mmol, 100%).

IR (DCM cast film, v_{max} / cm^{-1}) 3275, 3063, 2955, 2871, 1688, 1538, 1455, 1439, 1385, 1268, 1176, 1134

^{1}H NMR (700 MHz, $CDCl_3$) δ_H 7.84 (1H, d, *J* = 5.3 Hz, NH), 7.37—7.27 (5H, m, H1, H2, H3), 5.97 (1H, s, NH), 5.34 (1H, d, *J* = 7.0 Hz), 5.09 (2H, s, H5), 4.51—4.42 (1H, m, H9), 4.36—4.27 (1H, m, H7), 3.72 (3H, s, H11), 3.35—3.25 (2H, m, H15), 2.42—2.32 (2H, m, H13, H14), 2.21—2.10 (1H, m, H12), 1.95—1.88 (1H, m, H12), 1.86—1.78 (1H,

m, H14), 1.78—1.71 (1H, m, H18), 1.71—1.65 (1H, m, H17), 1.56—1.47 (1H, m H17), 0.99—0.91 (6H, m, H19)

^{13}C NMR (175 MHz, $CDCl_3$) δ_C 179.7 (C16), 172.8 (C8), 172.1 (C10), 156.1 (C6), 136.4 (C4), 128.5 (C2), 128.1 (C3), 128.0 (C1), 66.9 (C5), 53.4 (C7), 52.4 (C11), 51.7 (C9), 42.3 (C17), 40.5 (C15), 38.4 (C13), 32.9 (C12), 28.6 (C14), 24.6 (C18), 22.9 (H19), 22.1 (H19)

OR: $[\alpha]_D^{26}$ = -12.51 (*c* = 0.78, DCM)

HRMS: (ESI) (ESI) Calcd for $C_{22}H_{31}N_3NaO_6$ $[M + Na]^+$ 456.2105, found 456.2101

N^2-[(Benzyloxy)carbonyl]-N-{(2S)-1-hydroxy-3-[(3S)-2-oxo-3-pyrrolidinyl]-2-propanyl}-L-leucinamide **(4)**

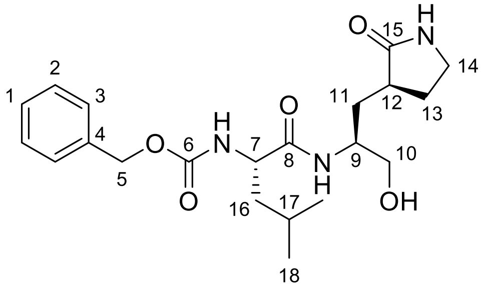

This compound was synthesized based on a literature procedure[2]. **(3)** (0.167 g, 0.385 mmol, 1.0 equiv) was deposited in a flame-dried round bottom flask under argon and dissolved in 2.31 mL of dry THF. To the RM was then added $LiBH_4$ (2.0 M in THF, 0.578 mL, 1.156 mmol, 3.0 equiv) dropwise. Addition causes RM to turn bright yellow in colour. Continued addition eventually results in fading of yellow colour. Gas evolution occurred. After gas evolution ceased, 1.16 mL of dry MeOH was added dropwise. This was followed by a second gas evolution event. RM was allowed to stir at rt for 1.5 h

under a blanket of argon. Reaction mixture was worked up by quenching with 1 M HCl until pH 1—2. The reaction mixture was then concentrated on rotovap and the resulting residue was dissolved in EtOAc and brine. The layers were separated and the EtOAc layer was dried over Na_2SO_4. Concentration and co-evaporation with Et_2O furnishes a white foam as the product (R_f = 0.14, 5/95 MeOH/EtOAc) (0.14 g, 0.345 mmol, 90%) with no further purification required.

IR (DCM cast film, v_{max} / cm^{-1}) 3282, 3065, 2955, 2871, 1686, 1539, 1456, 1442, 1386, 1369, 1263, 1173

^{1}H NMR (500 MHz, $CDCl_3$) δ_H 7.79—7.63 (1H, m, NH), 7.40—7.27 (5H, m, H1, H2, H3), 6.51—6.00 (1H, m, NH), 5.74—5.41 (1H, m, NH), 5.09 (2H, s, H5), 4.32—4.16 (1H, s, H7), 4.04—3.90 (1H, s, H9), 3.73—3.40 (3H, m, H10, OH), 3.34—3.18 (2H, m, H14), 2.55—2.25 (2H, m, H12, H13), 2.06—1.94 (1H, m, H11), 1.85—1.73 (1H, m, H13), 1.73—1.56 (3H, m, H11, H16, H17), 1.58—1.44 (1H, m, H16), 1.01—0.84 (6H, m, H18)

^{13}C NMR (125 MHz, $CDCl_3$) δ_C 181.0 (C15), 173.7(C8), 156.2 (C6), 136.4 (C4), 128.5 (C2), 128.1 (C3), 128.0 (C1), 66.9 (C5), 66.1 (C10), 53.9 (C7), 51.3 (C9), 42.3 (C16), 40.6 (C14), 38.6 (C12), 32.1 (C11), 28.9 (C13), 24.8 (C17), 23.0 (C18), 22.0 (C18)

OR: $[\alpha]_D^{26}$ = -21.20 (*c* = 1.05, DCM)

HRMS: (ESI) Calcd for $C_{21}H_{32}N_3O_5$ $[M + H]^+$ 406.2336, found 405.2264

(2S)-2-((S)-2-(((benzyloxy)carbonyl)amino)-4-methylpentanamido)-3-(2-oxopyrrolidin-3-yl)propanal **(GC373)**

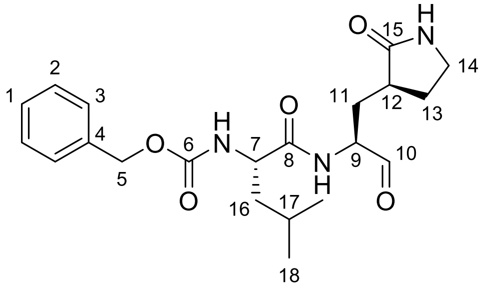

This compound was synthesized based on a literature procedure[2]. **(4)** (0.319 g, 0.786 mmol, 1.0 equiv) was dissolved in 0.8 mL of dry DCM under argon. Dess—Martin periodinane (0.500 g, 1.179 mmol, 1.5 equiv) was then added at 0 °C. The reaction mixture was capped under a blanket of argon and allowed to warm to rt. The reaction was then allowed to stir at rt for 3 h. Reaction mixture was then quenched with the addition of 3 mL of 10% $Na_2S_2O_3$ followed with stirring for 15 min. The layers were then separated and the DCM layer was washed sequentially with 10% $Na_2S_2O_3$ (1 x 3 mL), sat. $NaHCO_3$ (2 x 3 mL), H_2O (2 x 3 mL), and brine (2 x 3 mL). The DCM layer was then dried over Na_2SO_4 and concentrated to furnish a yellow residue. The TLC of this crude is very messy (extensive streaking) but the product was successfully purified *via* flash column chromatography on silica using an eluent system of 2.5/97.5 MeOH/EtOAc. Elution of product was monitored by TLC and $KMnO_4$ staining (R_f = 0.14, 2.5/97.5 MeOH/EtOAc). Concentration of product fractions furnishes an oil that solidifies to a light yellow foam upon co-evaporation with Et_2O (0.206 g, 0.511 mmol, 65%).

IR (DCM cast film, v_{max} / cm^{-1}) 3287, 3064, 2957, 2871, 1691, 1535, 1456, 1440, 1386, 1369, 1265, 1119

Please note that this compound exists as a mixture of diastereomers due to rapid epimerization at the alpha carbon of alpha-amido aldehydes. Although the ^{1}H NMR spectra does not appear to readily show this, some ^{13}C NMR signals do appear separated and have been noted as pairs where appropriate.

^{1}H NMR (500 MHz, $CDCl_3$) δ_H 9.51 (1H, app d, J = 33.9 Hz, H10 of diastereomers), 8.42 (1H, app dd, J = 77.0, 4.62 Hz, NH of diastereomers), 7.42—7.27 (5H, m, H1, H2, H3), 6.18 (1H, app d, J = 77.0 Hz, NH of diastereomers), 5.45 (1H, app dd, J = 36.5, 8.25 Hz, NH of diastereomers), 5.16—5.00 (2H, d, J = 9.5 Hz, H5), 4.55—4.07 (2H, m, H7, H9), 3.40—3.17 (2H, m, H14), 2.58—2.20 (2H, m, H12, H13), 2.14—2.00 (1H, m, H11), 2.00—1.88 (1H, m, H11), 1.88—1.80 (1H, m, H13), 1.79—1.62 (2H, m, H16, H17), 1.62—1.44 (1H, m, H16), 1.03—0.84 (6H, m, H18)

^{13}C NMR (125 MHz, $CDCl_3$) δ_C 200.8/199.7 (C10), 180.2/180.1 (C15), 173.7/173.4 (C8), 156.1 (C6), 136.4 (C4), 128.6/128.5 (C2), 128.2/128.1 (C3), 128.0 (C1), 67.0/66.9 (C5), 57.9/57.0 (C7), 53.7/53.6 (C9), 42.3/41.9 (C16), 40.6 (C14), 38.2/37.7 (C12), 29.7/29.1 (C11), 28.8/28.3 (C13), 24.8 (C17), 23.0/23.0 (C18), 22.0/21.9 (C18)

OR: $[\alpha]_D^{26}$ = -2.93 (c = 0.43, DCM)

HRMS: (ESI) Calcd for $C_{21}H_{30}N_3O_5$ $[M + H]^+$ 404.2180, found 404.2173

Sodium (2S)-2-((S)-2-(((benzyloxy)carbonyl)amino)-4-methylpentanamido)-1-hydroxy-3-(2-oxopyrrolidin-3-yl)propane-1-sulfonate **(GC376)**

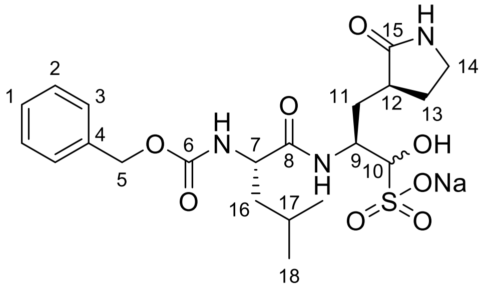

This compound was synthesized based on a literature procedure[2]. **GC373** (0.030 g, 0.074 mmol, 1.0 equiv) was deposited in a vial and dissolved in 0.296 mL of dry EtOAc and 0.178 mL of absolute EtOH. To this was added 1 M $NaHSO_3$ (0.074 mL, 0.074 mmol, 1.0 equiv). The reaction vessel was then capped and stirred at 50 °C for 3 h. Afterwards, the mixture was filtered to remove solids and the solids were thoroughly washed with additional volumes of EtOH. The combined washings were dried over Na_2SO_4 and filtered again. Concentration of the filtrate furnishes a yellow oil. To this oil was added 0.5 mL of Et_2O, causing a white solid to crash out after mixing. The mixture was then centrifuged and the Et_2O was removed. This step was repeated once more. The resultant white solid was then treated with Et_2O (0.444 mL) and EtOAc (0.222 mL). Mixing for 5 min followed by centrifuge and removal of solvent furnishes the white bisulfite adduct as the product. The material was dried on hi-vac overnight to remove residual solvent. A check by LCMS shows the desired adduct as the major product (0.025 g, 0.050 mmol, 68%) and was used without further purification.

IR (H_2O cast film, v_{max} / cm^{-1}) 3291, 3090, 3066, 3035, 2957, 2872, 1674, 1527, 1455, 1387, 1368, 1216

Please note that this compound exists as a mixture of diastereomers due to rapid epimerization at the alpha carbon of alpha-amido aldehydes. Although the ^{1}H NMR spectra does not appear to readily show this, some ^{13}C NMR signals do appear separated and have been noted as pairs where appropriate.

^{1}H NMR (500 MHz, DMSO-d6) δ_H 7.65—7.56 (1H, m NH), 7.50—7.42 (1H, m NH), 7.39—7.31 (5H, m, H1, H2, H3), 5.38 (1H, d, *J* = 4.5 Hz, NH), 5.24 (1H, d, *J* = 4.5 Hz, OH), 5.07—4.96 (2H, m, H5), 4.25—3.79 (3H, m, H7, H9, H10), 3.14—3.07 (1H, m, H14), 3.04—2.98 (1H, m, H14), 2.22—2.04 (2H, m, H12, H13), 2.03—1.52 (4H, m, H11, H13, H17), 1.50—1.40 (2H, m, H16), 0.85 (6H, ddd, *J* = 12.4, 8.9, 4.4 Hz, H18)

^{13}C NMR (125 MHz, DMSO-d6) δ_C 179.0 (C15), 171.8 (C8), 156.0 (C6), 137.1 (C4), 128.2/128.2 (C2), 127.6 (C3), 127.6/127.6 (C1), 84.4/83.7 (C10), 65.3 (C5), 53.6/53.5 (C9), 49.1/48.6 (C7), 40.5 (C16), 40.7/40.4 (C14), 37.8/37.7 (C12), 31.8 (C11), 27.5/27.3 (C13), 24.2/24.2 (C17), 23.1/23.0 (C18), 21.4/21.3 (C18)

OR: $[\alpha]_D^{26}$ = -37.38 (*c* = 0.31, H_2O)

HRMS: (ESI) Calcd for $C_{21}H_{32}N_3O_8S$ $[M + H]^+$ 486.1905, found 486.1896

Synthesis of ^{13}C Labelled GC373

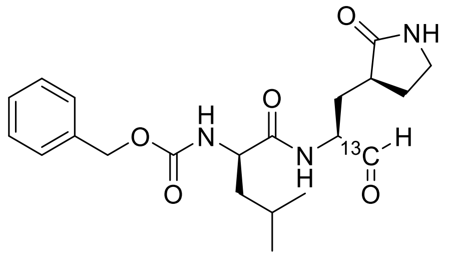

GC373 - ^{13}C

Synthesis of ^{13}C labelled GC373 was carried out *via* the above documented methods. All characterization data was in agreement as described above.

HRMS: (ESI) Calcd for $C_{20}{}^{13}CH_{30}N_3O_5$ $[M + H]^+$ 405.2214, found 405.2215

FRET Substrate Peptide Synthesis

General 2-Chlorotrityl Chloride Resin Loading Procedure

2-Chlorotrityl chloride resin was transferred to a SPPS vessel and washed with dry CH_2Cl_2 (2 × 10 mL) and then dry DMF (2 × 10 mL) for one min each, and then bubbled under Ar in dry DMF (10 mL) for 10 min. The desired Fmoc-protected amino acid (1.0 equiv, based on desired resin loading) and DIPEA (5.0 equiv) were suspended in 10 mL of a 50/50 mixture of dry CH_2Cl_2/DMF. This solution was bubbled under Ar for 2.5 h to load the desired amino acid onto the solid support, continually topping up the CH_2Cl_2 to maintain an approximately 10 mL volume. To end cap any remaining trityl groups, dry MeOH was added to the vessel (0.8 mL per gram of resin) and bubbled under Ar for 15 minutes. After draining, the resin was washed with dry DMF (3 × 10 mL), dry CH_2Cl_2 (3 × 10 mL), and then with dry MeOH (3 × 10 mL) for one min each. The resin was dried thoroughly and then stored at –20 °C under Ar.

General Automated SPPS Elongation Method

All peptides were synthesized on a PreludeX (Gyros protein technologies). SPPS was carried out on a 0.1 mmol scale using Fmoc chemistry on 2-chlorotrityl resin (0.8 mmol/g). Commercially available Fmoc-protected amino acids were loaded on the

peptide synthesizer as 0.2 M solutions in DMF. All amino acids were coupled using 1-[Bis(dimethylamino)-methylene]-1H-1,2,3-triazolo[4,5-b]pyridinium 3-oxide hexafluorophosphate (HATU) as the activating agent with a coupling time of 1 h. Fmoc residues were deprotected using a 20% solution of piperidine in DMF.

General Method for Cleavage of Peptide from Resin

Resin-bound analogue was suspended in 95/2.5/2.5 TFA/TIPS/H_2O with shaking for 2-3 h. The resin was removed *via* filtration through glass wool, rinsed with TFA, and the solution concentrated *in vacuo*. Cold diethyl ether (2 × 5 mL) was added to triturate the crude residue. The diethyl ether was decanted and briefly centrifuged for 3 minutes at 13000 rpm to pellet any residual peptide. The ether was removed and the peptide pellet was then dried thoroughly by centrifugation in a vacuum centrifuge for 5 minutes. The pellet and triturated crude residue were pooled together and dissolved in 0.1% aqueous TFA.

HPLC Purification Method

The FRET peptide substrate was purified using a C_{18} RP-HPLC column with aqueous 0.1% TFA (solvent A) and 0.1% TFA in acetonitrile (solvent B) as eluents. The analytical purification method used was: 0 – 3 min 10% B, 3 – 4.5 min 10% – 25% B, 4.5 – 14.5 min 25% – 40% B, 14.5 – 17 min 40% – 90% B, 17 – 19.5 min 95% B, 19.5 – 20.5 min 95% – 10% B, 20.5 – 30 min 10% B.

Synthesis of FRET Peptide Substrate

Fmoc-L-Arg(Pmc)-OH was loaded onto 2-chlorotrityl chloride resin (0.18 mmol/g) using the aforementioned general procedure. The resin was elongated using automated SPPS, introducing amino acids in the following order: Fmoc-Tyr(NO2)-OH, Fmoc-Gly-OH, Fmoc-Ser(OtBu)-OH, Fmoc-Gln(Trt)-OH, Fmoc-Leu-OH, Fmoc-Thr(tBu)-OH, Fmoc-Val-OH, Fmoc-Ser(OtBu)-OH, and Boc-Abz-OH with the final N-terminal Boc group being left on the peptide. The peptide was then cleaved off the resin using the aforementioned procedure, and purified using a Vydac Si C18 RP-HPLC semi-preparative column (300 Å, 5 μM, 10 × 250 mm) and HPLC method A, with the desired peptide eluting at 16.2 minutes. The HPLC fractions were pooled and lyophilized to produce the peptide as a yellow powder. The peptide was analyzed using HRMS (ESI) calcd for $C_{50}H_{77}N_{15}O_{18}$ $[M + 2H]^{2+}$ 587.7780, found 587.7781.

GC373 NMR Binding Assay

NMR Samples

NMR samples were first prepared by dialyzing SARS-CoV-2 M^{pro} enzyme to exchange buffers (target buffer: D_2O, 50 mM phosphate, pD 7.5 with 20 mM DTT) by spin filtration (Amicon micro-spinfilter, 10 kDa cutoff). A 50 μL solution of 2.6 mg/mL enzyme was added to the spin filter and diluted to 300 μL, then spun at 6600 g for 18 min. This was repeated an additional 2 times, and the sample was then made up to 300 μL in volume and transferred to the NMR tube. Samples of enzyme in the presence of inhibitor were prepared by administering an additional 1.5 μL of ^{13}C labelled GC373 solution (20 mM in DMSO) to the aforementioned enzyme sample. Sample data was

acquired in 5 mm Varian specific D_2O susceptibility matched microcell (i.e. BMS-00V) Shigemi NMR tubes purchased from Wilmad Lab-glass Inc. All NMR solvents were purchased from Sigma Aldrich. NMR tubes were washed between runs using 5 rinses of D_2O, and then inverted to air dry overnight.

Small volume additions (e.g. inhibitor added to enzyme) to samples in Shigemi NMR tubes were done by adding to the top of the outer NMR tube with the inner plunger removed, and then carefully tapping the sample tube in an almost horizontal position while rotating to break the surface tension and allow sample liquid to flow up the tube. The sample volume was allowed to travel up until making contact with the additional material. The tube was then repeatedly whipped downward to move the material to the bottom. This was repeated several times so that additions were rinsed down into the microcell ensuring proper mixing.

NMR Spectroscopy

NMR experiments were collected at 16.45 T (i.e. 700 MHz) using a 4-channel 'VNMRS' (Varian/Agilent) NMR spectrometer (VNMRJ 4.2 patch110 software) with an Agilent 7620 automatic sample handling system. A 5mm triple resonance cryogenically cooled (20 K) ^{1}H direct-detection (i.e. with $^{13}C^{15}N$ on the outer coil) probe was utilized for all experiments. The probe had cooled preamplifiers on the 1H and 13C detection channels. The sample temperature was calibrated to 27°C using methanol[3].

All spectra were run "locked" on the ^{2}H resonance signal and chemical shifts were referenced using the residual proton ^{1}HOD signal position[4] (i.e. 4.7 ppm) prior to saturation. One dimensional ^{1}H data were acquired using presaturation[5,6] for residual

^{1}HOD solvent suppression, followed by a 90° excitation pulse and data acquisition. The saturation carrier position amplitude were manually optimized using a position sweep array and based on the calibrated high-power pulse, respectively. Saturation was applied directly on the water resonance (i.e. depending on water suppression efficacy and the ability to avoid analog to digital and/or receiver overloads with sufficient gain for data acquisition). The saturation was applied with a gammaB_1 induced field strength of ~80 Hz[7], and a duration of 2 seconds. The 90° pulse width was determined after tuning and matching each sample, using one-pulse nutation optimization[8]. Other specific 1D acquisition parameter settings were: sweep width of 14044 Hz, acquisition time 2.5 seconds, with 70224 total (i.e. real plus imaginary) data points.

A gradient-selected phase-sensitive two dimensional ^{1}H,^{13}C-heteronuclear single quantum correlation (HSQC)[9–11] with adiabatic inversion, refocusing and decoupling ^{13}C pulses (i.e. gHSQCAD Varian/Agilent "ChemPack", Krish Krishnamurthy) was used for all experiments. The HSQC spectra were acquired with ^{1}H and ^{13}C sweep widths (and recorded points) of 8389 Hz (~12 ppm) and 38722 Hz (220 ppm), respectively (1678 total directly detected and 32 complex indirectly detected points, also respectively). A gradient stabilization delay of 500 µs was used and 146 Hz J_{HC} applied for INEPT transfers. Adiabatic inversion/recovery ^{13}C pulses were applied at a 13.1 kHz induced field with a 600 us duration covering ~400 ppm. Adiabatic decoupling was applied at ~2.6 kHz induced field during the entire 100ms acquisition period. Carbon chemical shift referencing and carrier position (100 ppm) was based on indirect IUPAC ^{1}H referencing[12]. The carbon carrier position was moved (e.g. 80, 90, or 100 ppm) to

eliminate the possibility of peaks of interest near the carrier position being artifactual (i.e. quadrature “glitch”).

For processing of NMR data, all dimensions were zero-filled to twice the number of acquired points. A line-broadening apodization function of 0.25 Hz was applied for ^{1}H-1D spectra while a π/2 squared sine-bell weighting function was utilized for the 2D-HSQC (both dimensions). No linear prediction was applied nor was non-linear/non-uniform data acquisition utilized to avoid any possible artifacts in the resulting data. The final spectra were manually phased and baseline corrected.

References

1. Tian, Q. *et al.* An efficient synthesis of a key intermediate for the preparation of the rhinovirus protease inhibitor AG7088 via asymmetric dianionic cyanomethylation of N-Boc-L-(+)-glutamic acid dimethyl ester. *Tetrahedron Lett.* **42**, 6807–6809 (2001).
2. Galasiti Kankanamalage, A. C. *et al.* Structure-Guided Design and Optimization of Dipeptidyl Inhibitors of Norovirus 3CL Protease. Structure–Activity Relationships and Biochemical, X-ray Crystallographic, Cell-Based, and In Vivo Studies. *J. Med. Chem.* **58**, 3144–3155 (2015).
3. Raiford, D. S., Fisk, C. L. & Becker, E. D. Calibration of methanol and ethylene glycol nuclear magnetic resonance thermometers. *Anal. Chem.* **51**, 2050–2051 (1979).
4. Wishart, D. S. *et al.* 1H, 13C and 15N chemical shift referencing in biomolecular NMR. *J. Biomol. NMR* **6**, 135–140 (1995).

5. Hoult, D. . Solvent peak saturation with single phase and quadrature fourier transformation. *J. Magn. Reson.* **21**, 337–347 (1976).

6. Campbell, I. D., Dobson, C. M., Jeminet, G. & Williams, R. J. P. Pulsed NMR methods for the observation and assignment of exchangeable hydrogens: Application to bacitracin. *FEBS Lett.* **49**, 115–119 (1974).

7. Mao, X. & Chen, J. Radiation damping effects in solvent preirradiation experiments in NMR. *Chem. Phys.* **202**, 357–366 (1996).

8. Wu, P. S. C. & Otting, G. Rapid pulse length determination in high-resolution NMR. *J. Magn. Reson.* **176**, 115–119 (2005).

9. Bodenhausen, G. & Ruben, D. J. Natural abundance nitrogen-15 NMR by enhanced heteronuclear spectroscopy. *Chem. Phys. Lett.* **69**, 185–189 (1980).

10. Kong, X. M., Sze, K. H. & Zhu, G. Gradient and sensitivity enhanced multiple-quantum coherence in heteronuclear multidimensional NMR experiments. *J. Biomol. NMR* **14**, 133–140 (1999).

11. Stonehouse, J., Shaw, G. L., Keeler, J. & Laue, E. D. Minimizing Sensitivity Losses in Gradient-Selected 15N-1H HSQC Spectra of Proteins. *J. Magn. Reson. Ser. A* **107**, 178–184 (1994).

12. Markley, J. L. *et al.* Recommendations for the presentation of NMR structures of proteins and nucleic acids – IUPAC-IUBMB-IUPAB Inter-Union Task Group on the Standardization of Data Bases of Protein and Nucleic Acid Structures Determined by NMR Spectroscopy. *J. Biomol. NMR* **12**, 1–23 (1998).

Supplementary Figures

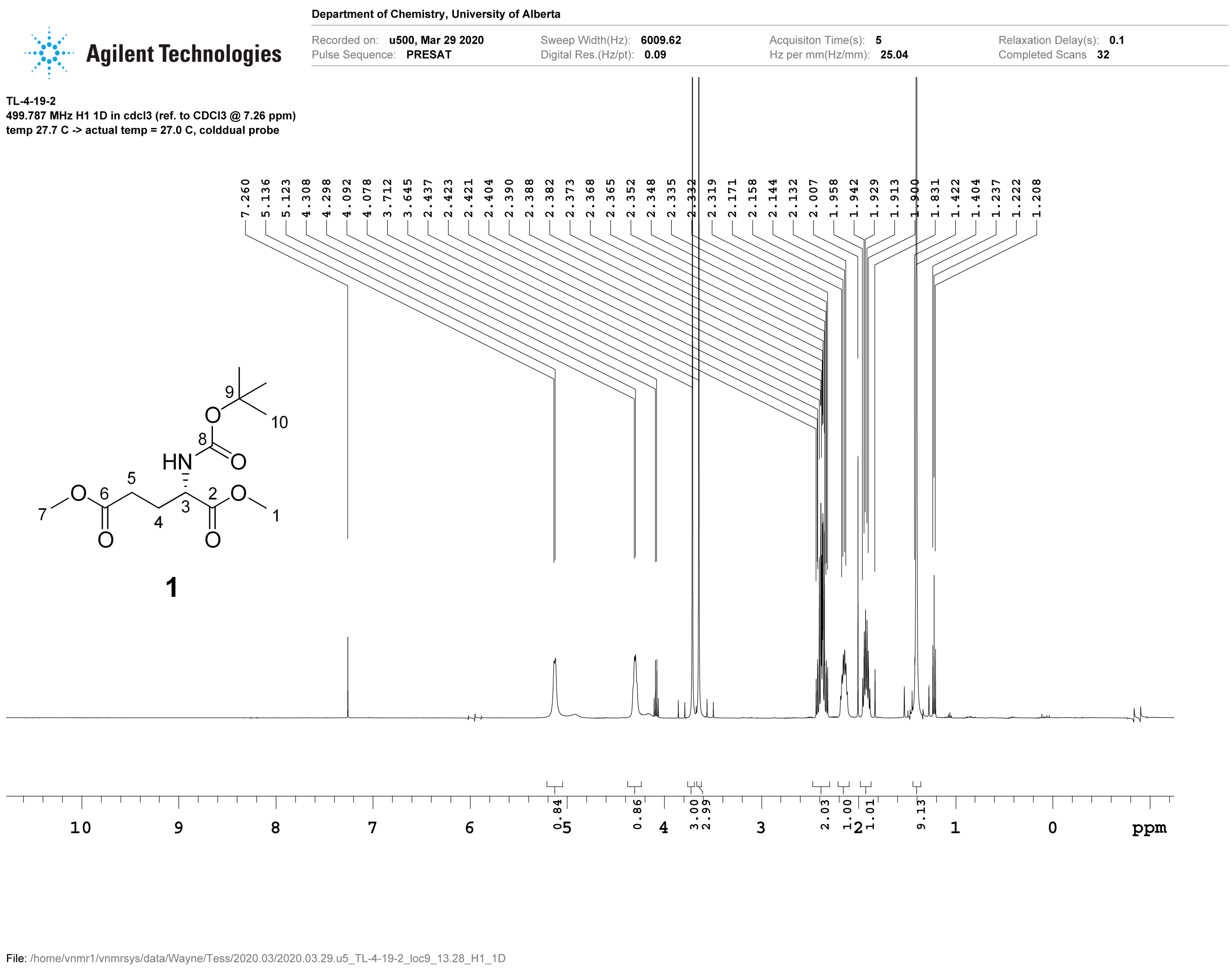

Supplementary Fig. 1. ^{1}H-NMR spectra and molecular structure of intermediate compound 1 . Identity and purity of product resulting from the synthetic steps described in the methods section above can be confirmed. Integration and chemical shift values correspond to values expected of the target compound.

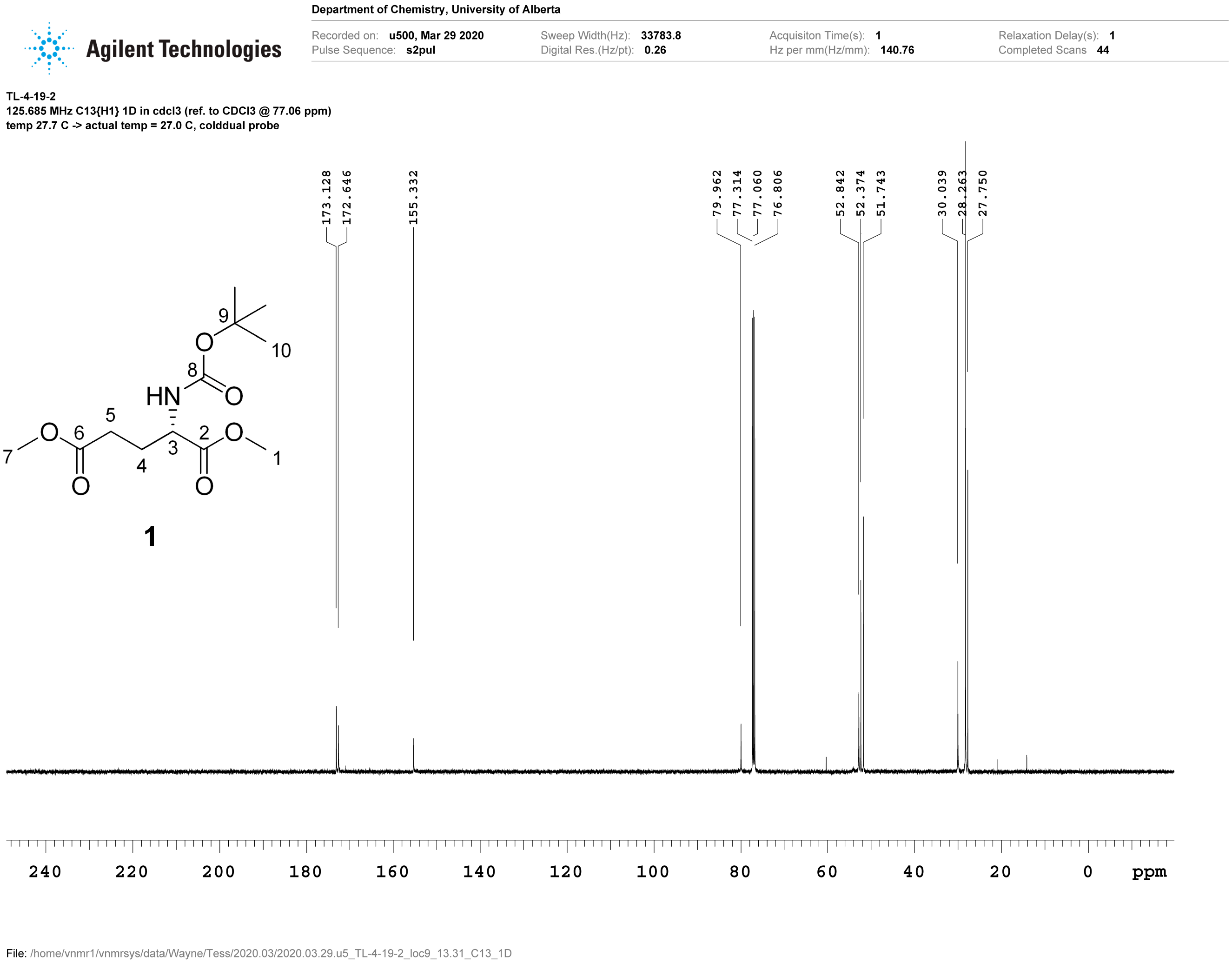

Supplementary Fig. 2. ^{13}C-NMR spectra and molecular structure of intermediate compound 1. Identity and purity of product resulting from the synthetic steps described in the methods section above can be confirmed. Chemical shift values correspond to values expected of the target compound.

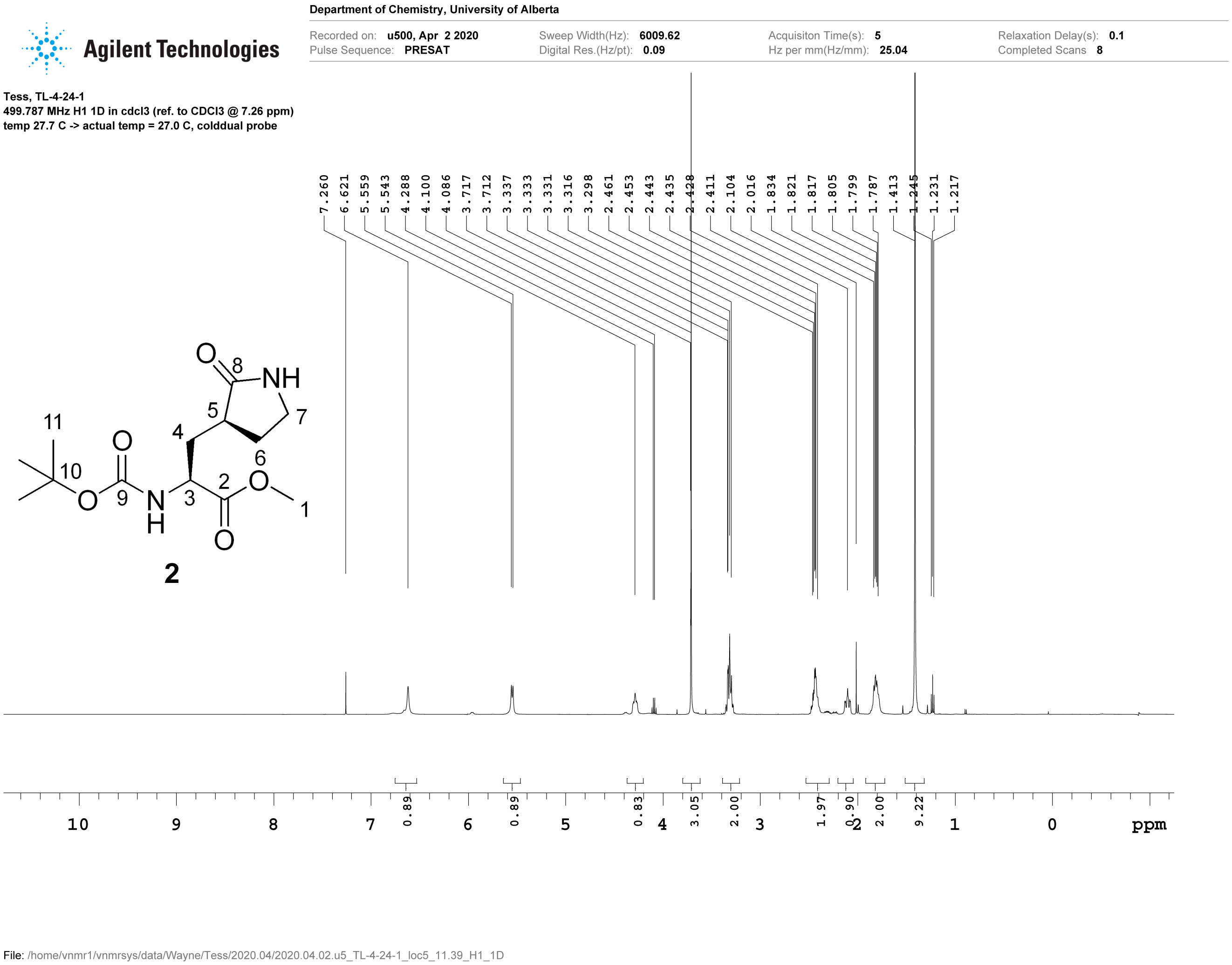

Supplementary Fig. 3. ^{1}H-NMR spectra and molecular structure of intermediate compound 2. Identity and purity of product resulting from the synthetic steps described in the methods section above can be confirmed. Integration and chemical shift values correspond to values expected of the target compound.

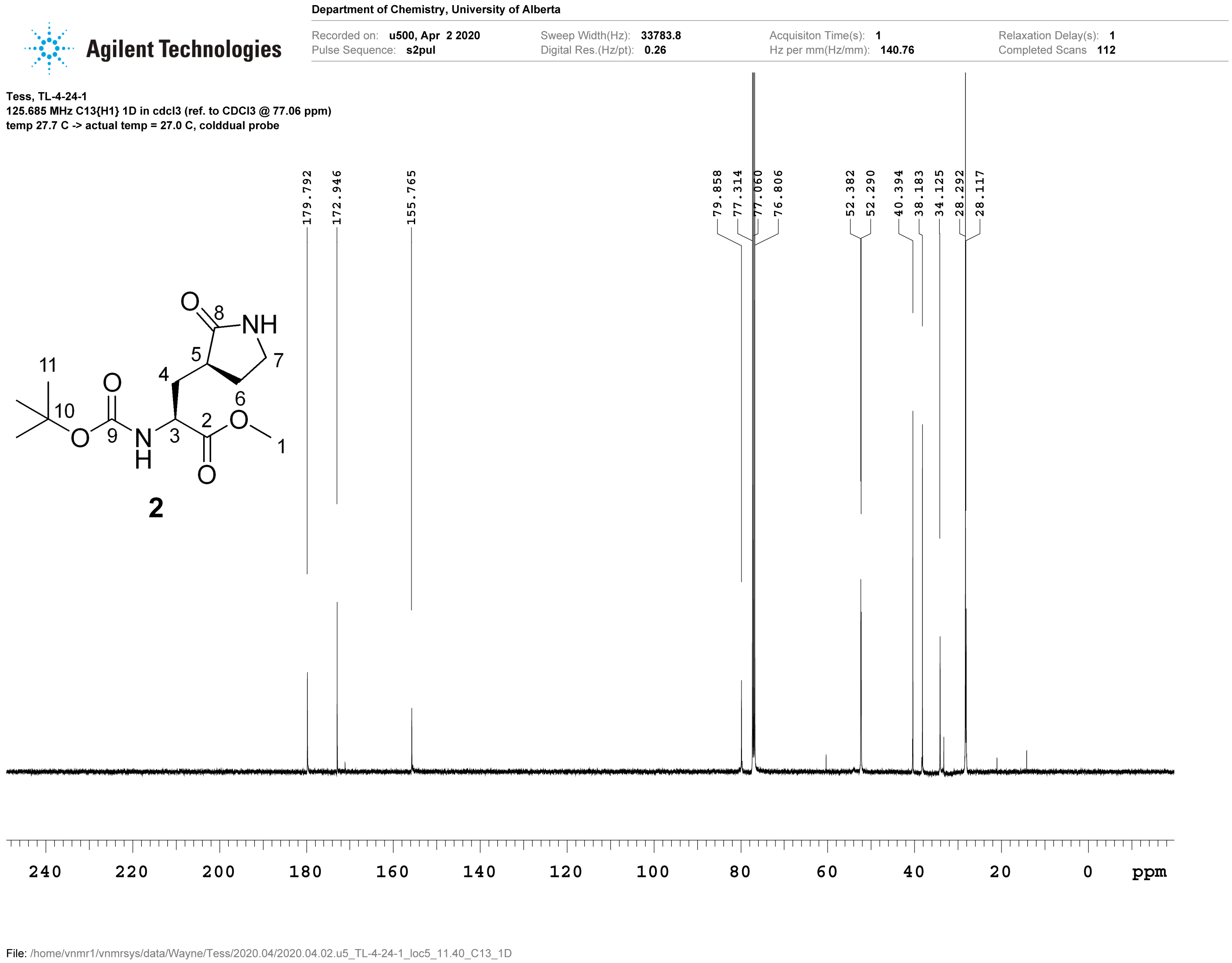

Supplementary Fig. 4. ^{13}C-NMR spectra and molecular structure of intermediate compound 2. Identity and purity of product resulting from the synthetic steps described in the methods section above can be confirmed. Chemical shift values correspond to values expected of the target compound.

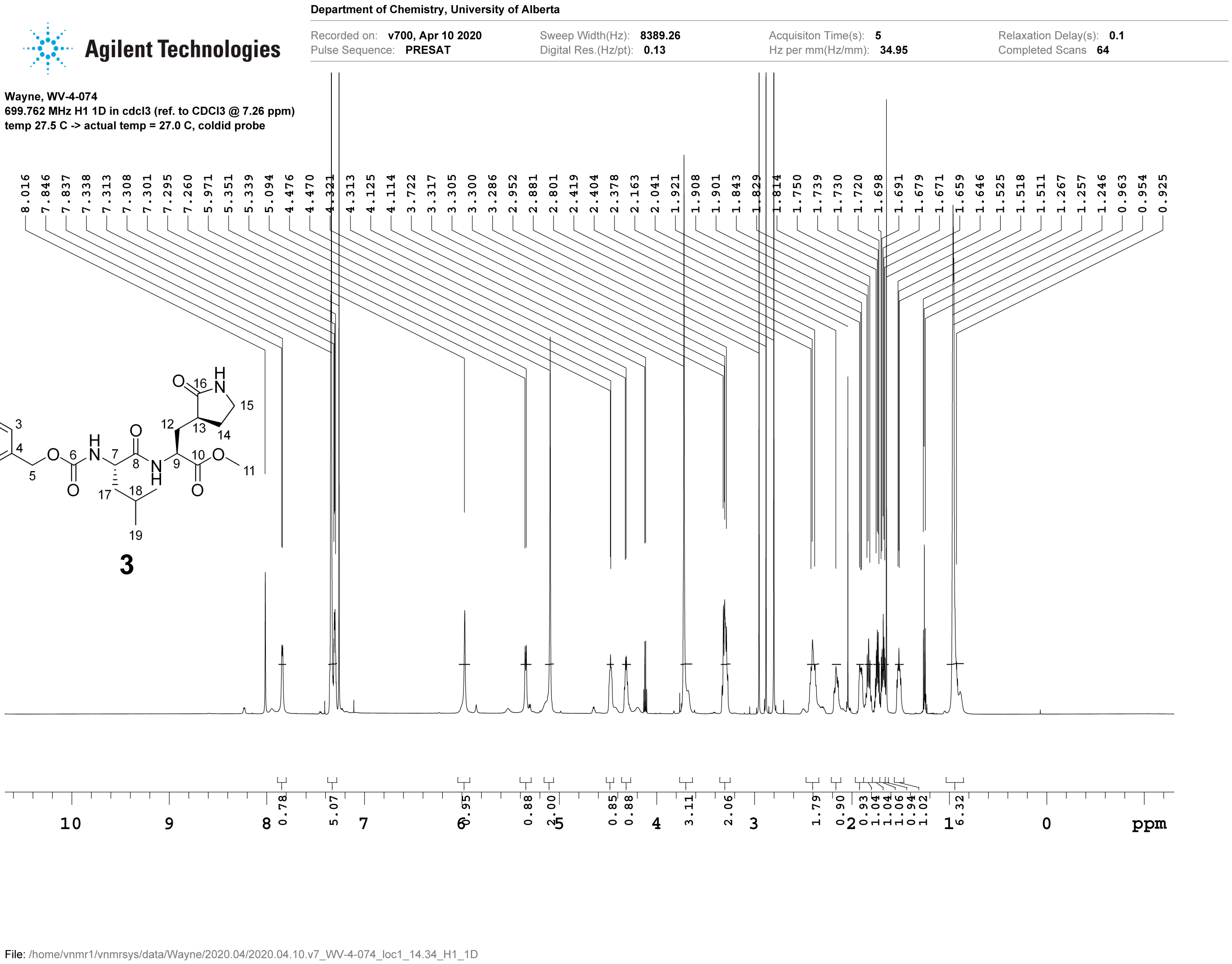

Supplementary Fig. 5. ^{1}H-NMR spectra and molecular structure of intermediate compound 3. Identity and purity of product resulting from the synthetic steps described in the methods section above can be confirmed. Integration and chemical shift values correspond to values expected of the target compound.

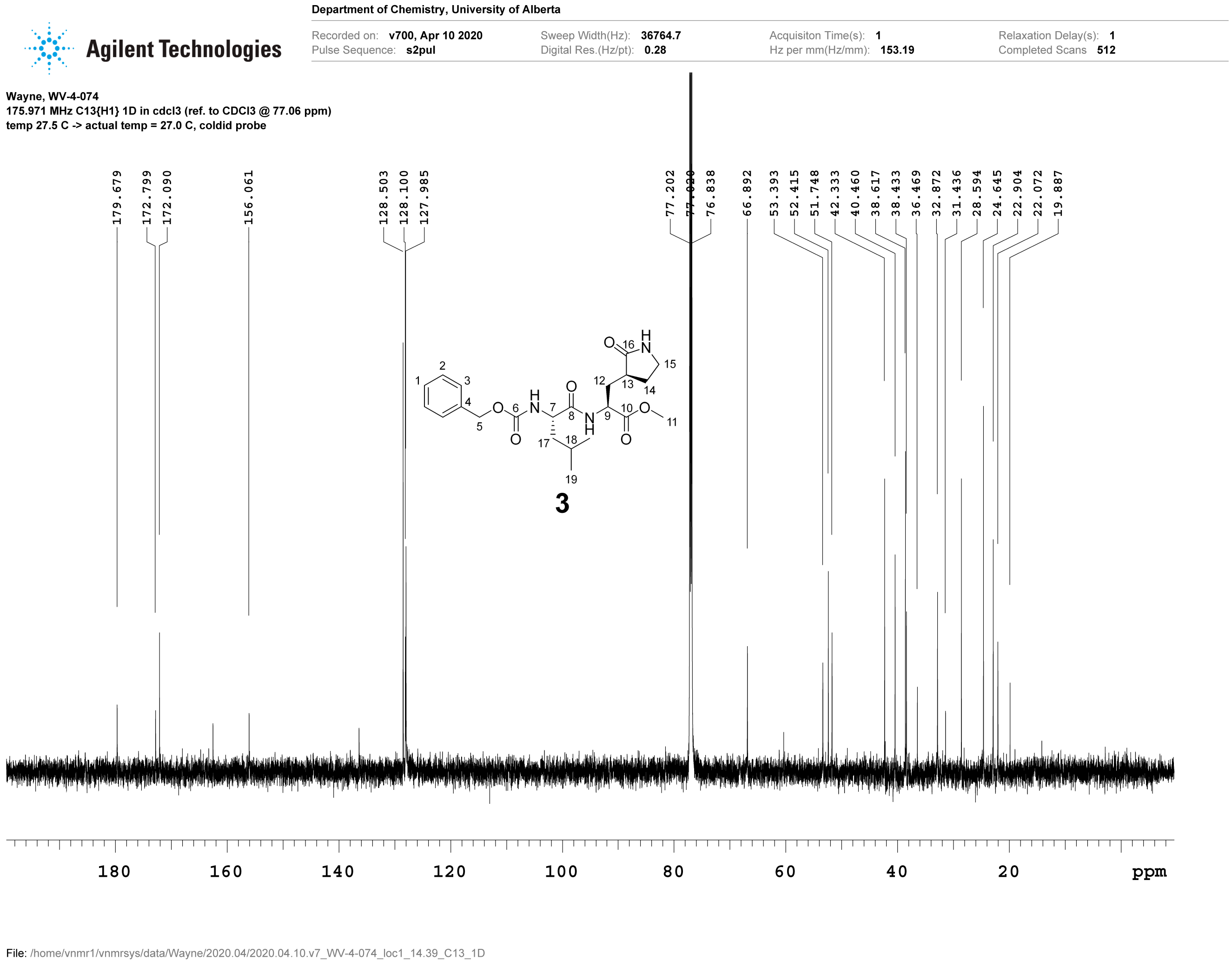

Supplementary Fig. 6. ^{13}C-NMR spectra and molecular structure of intermediate compound 3. Identity and purity of product resulting from the synthetic steps described in the methods section above can be confirmed. Chemical shift values correspond to values expected of the target compound.

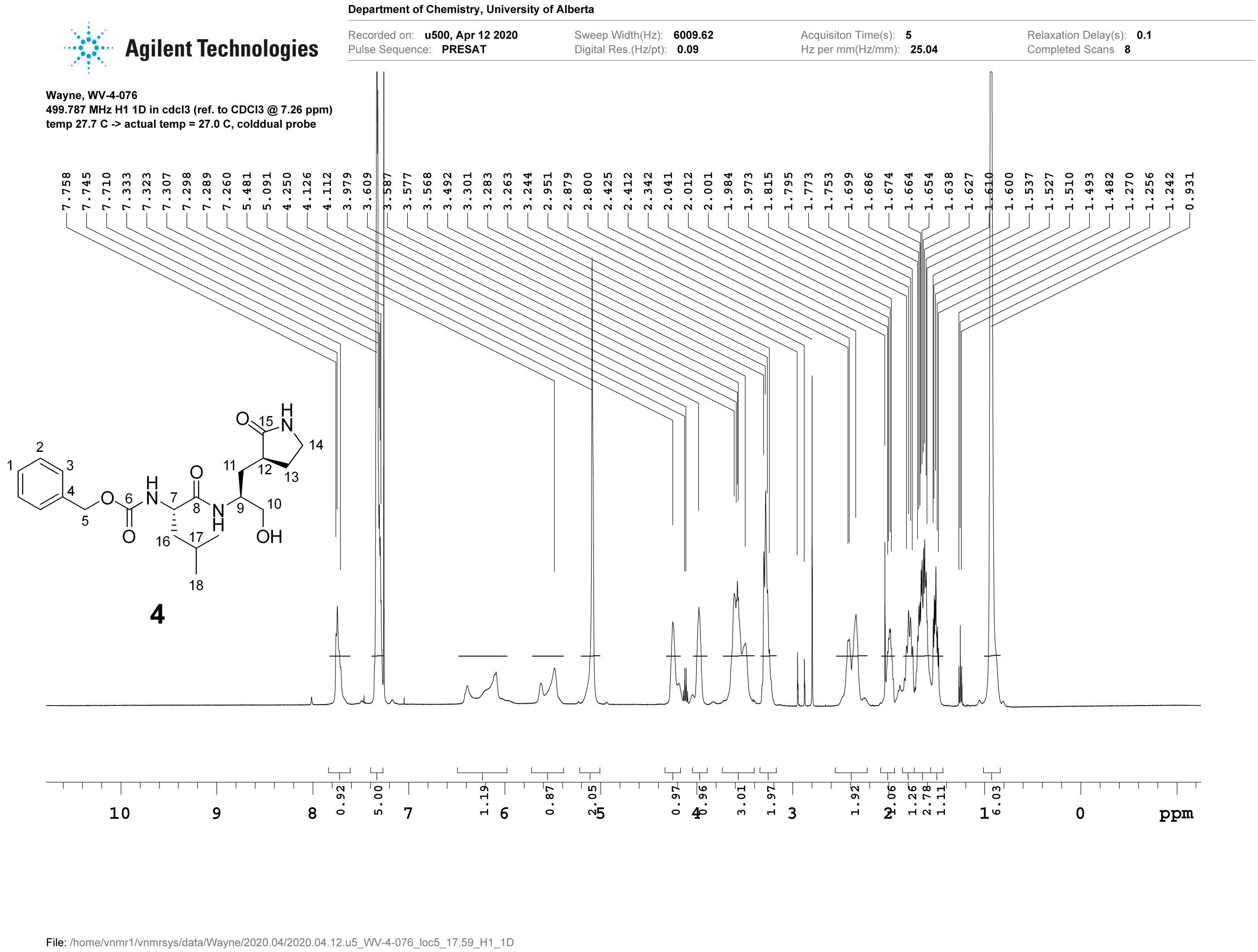

Supplementary Fig. 7. ^{1}H-NMR spectra and molecular structure of intermediate compound 4. Identity and purity of product resulting from the synthetic steps described in the methods section above can be confirmed. Integration and chemical shift values correspond to values expected of the target compound.

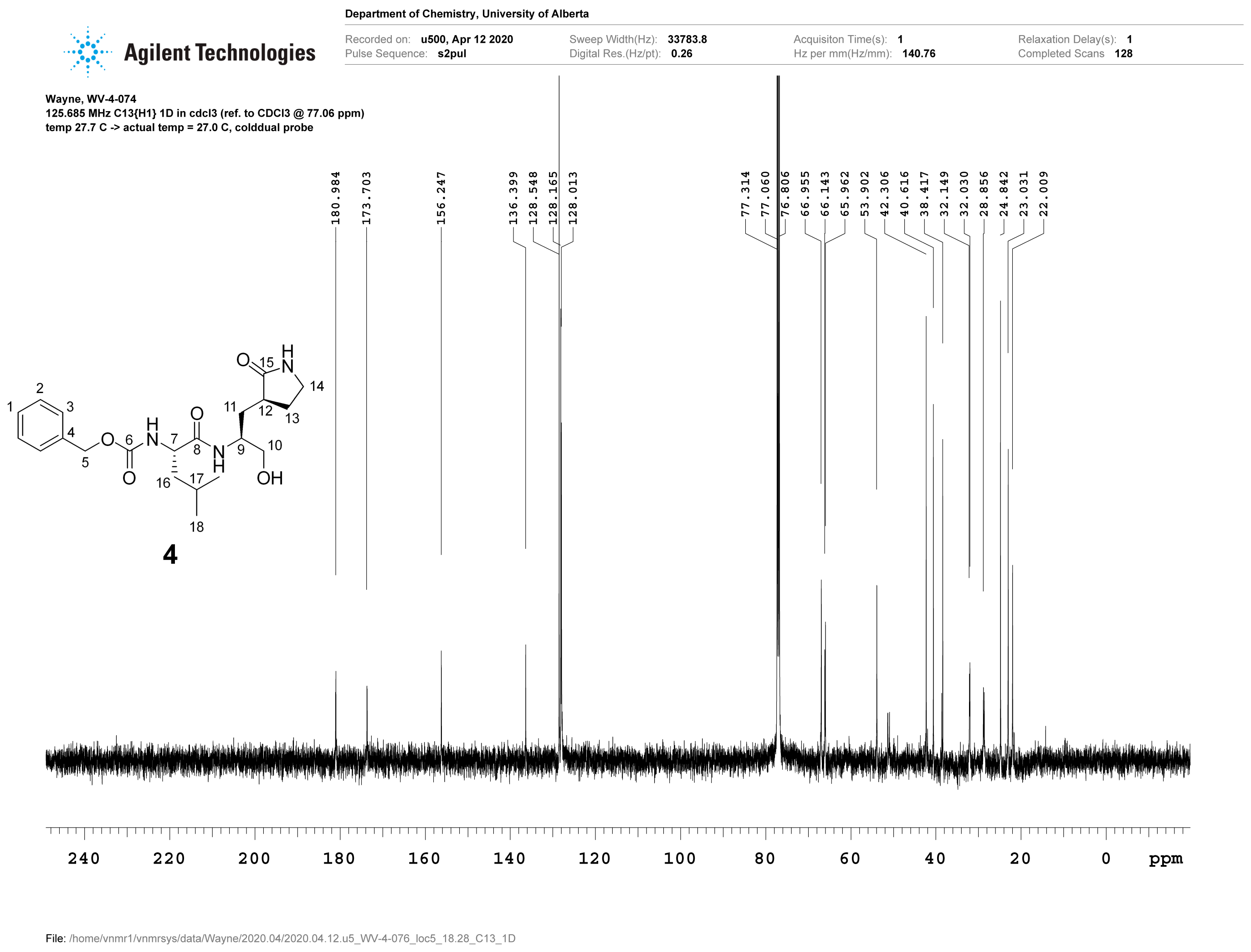

Supplementary Fig. 8. ^{13}C-NMR spectra and molecular structure of intermediate compound 4. Identity and purity of product resulting from the synthetic steps described in the methods section above can be confirmed. Chemical shift values correspond to values expected of the target compound.

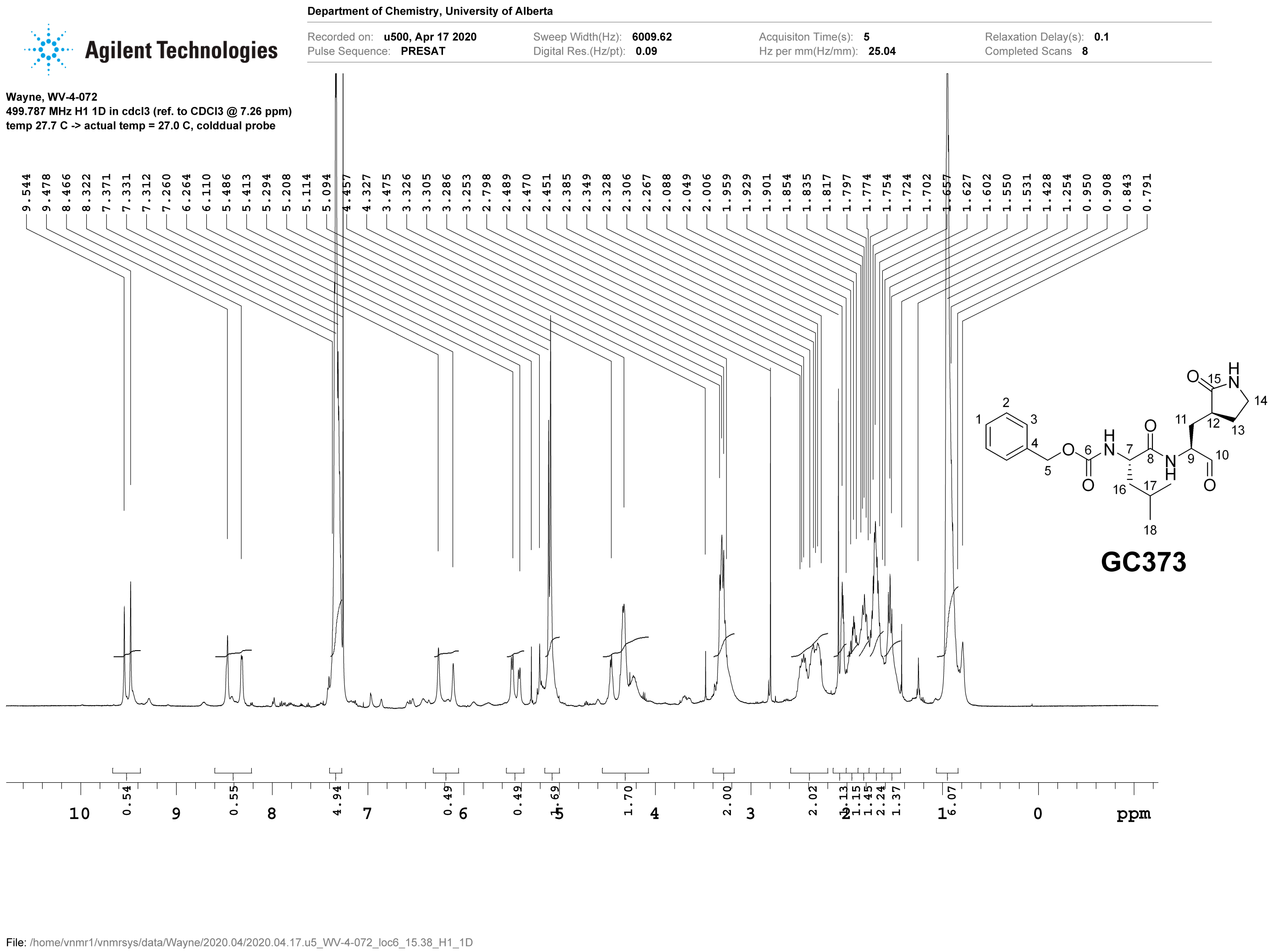

Supplementary Fig. 9. ^{1}H-NMR spectra and molecular structure of GC373. Identity and purity of product resulting from the synthetic steps described in the methods section above can be confirmed. Integration and chemical shift values correspond to values expected of the target compound.

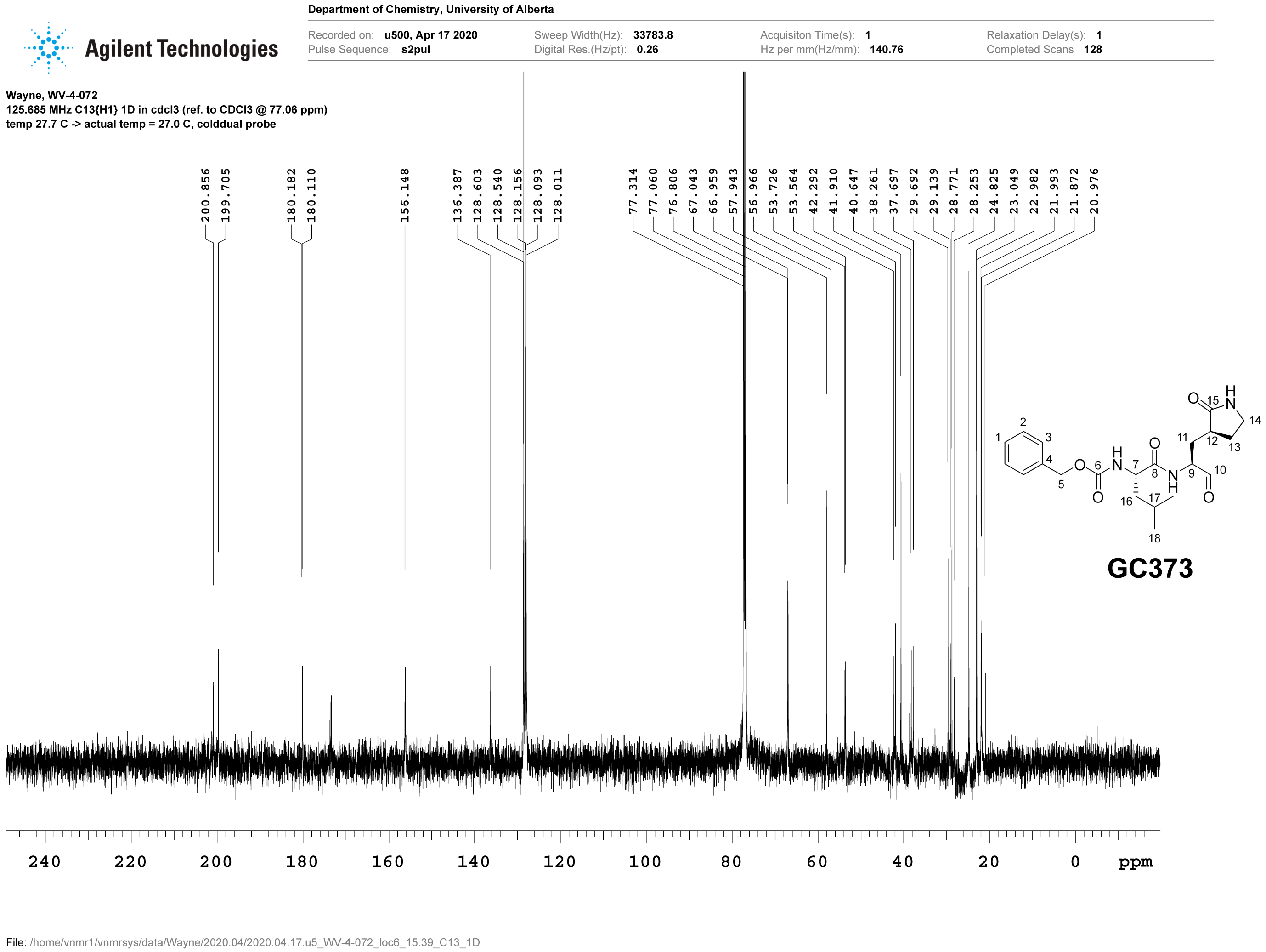

Supplementary Fig. 10. ^{13}C-NMR spectra and molecular structure of GC373. Identity and purity of product resulting from the synthetic steps described in the methods section above can be confirmed. Chemical shift values correspond to values expected of the target compound.

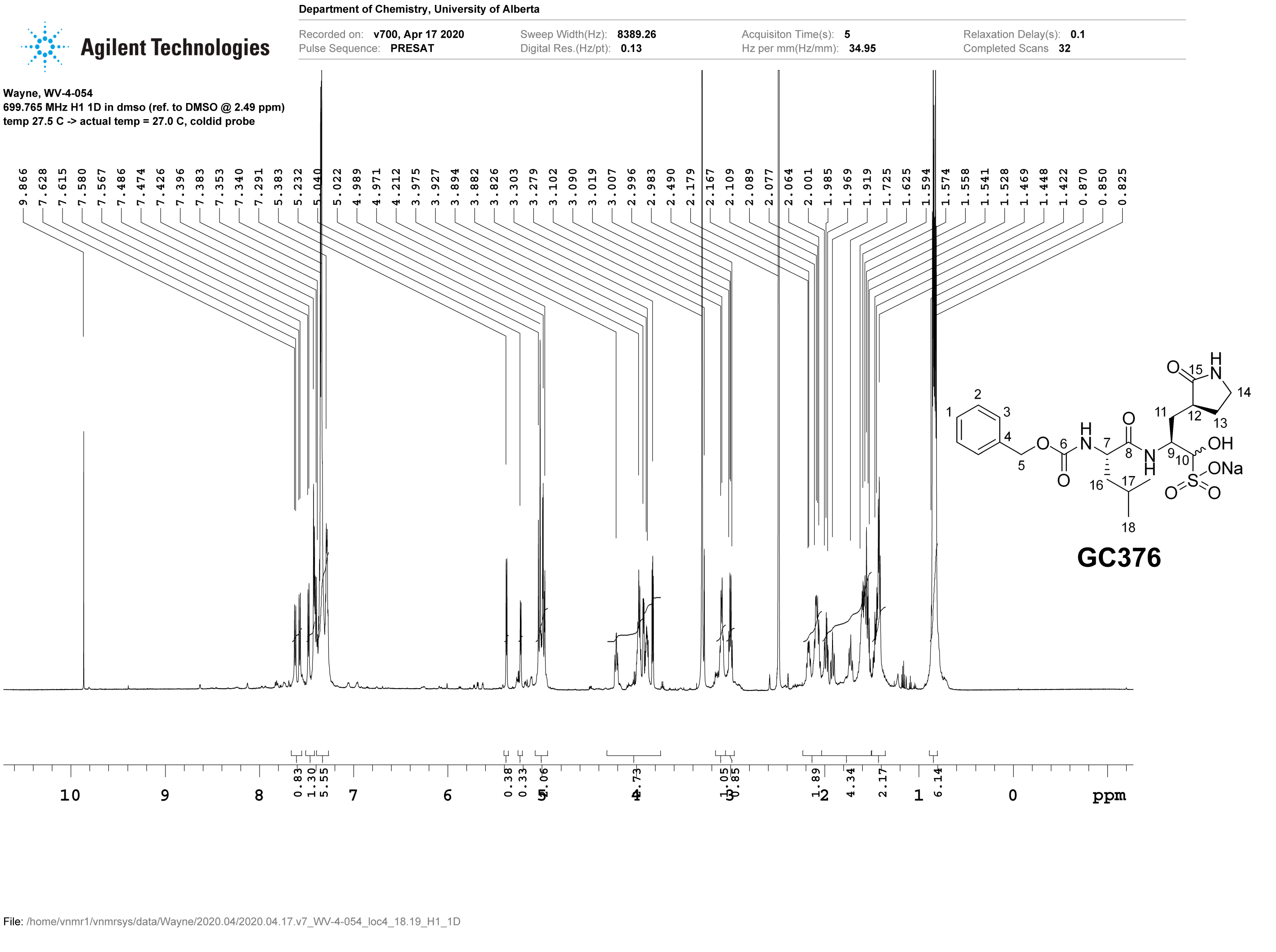

Supplementary Fig. 11. ^{1}H-NMR spectra and molecular structure of GC376. Identity and purity of product resulting from the synthetic steps described in the methods section above can be confirmed. Integration and chemical shift values correspond to values expected of the target compound.

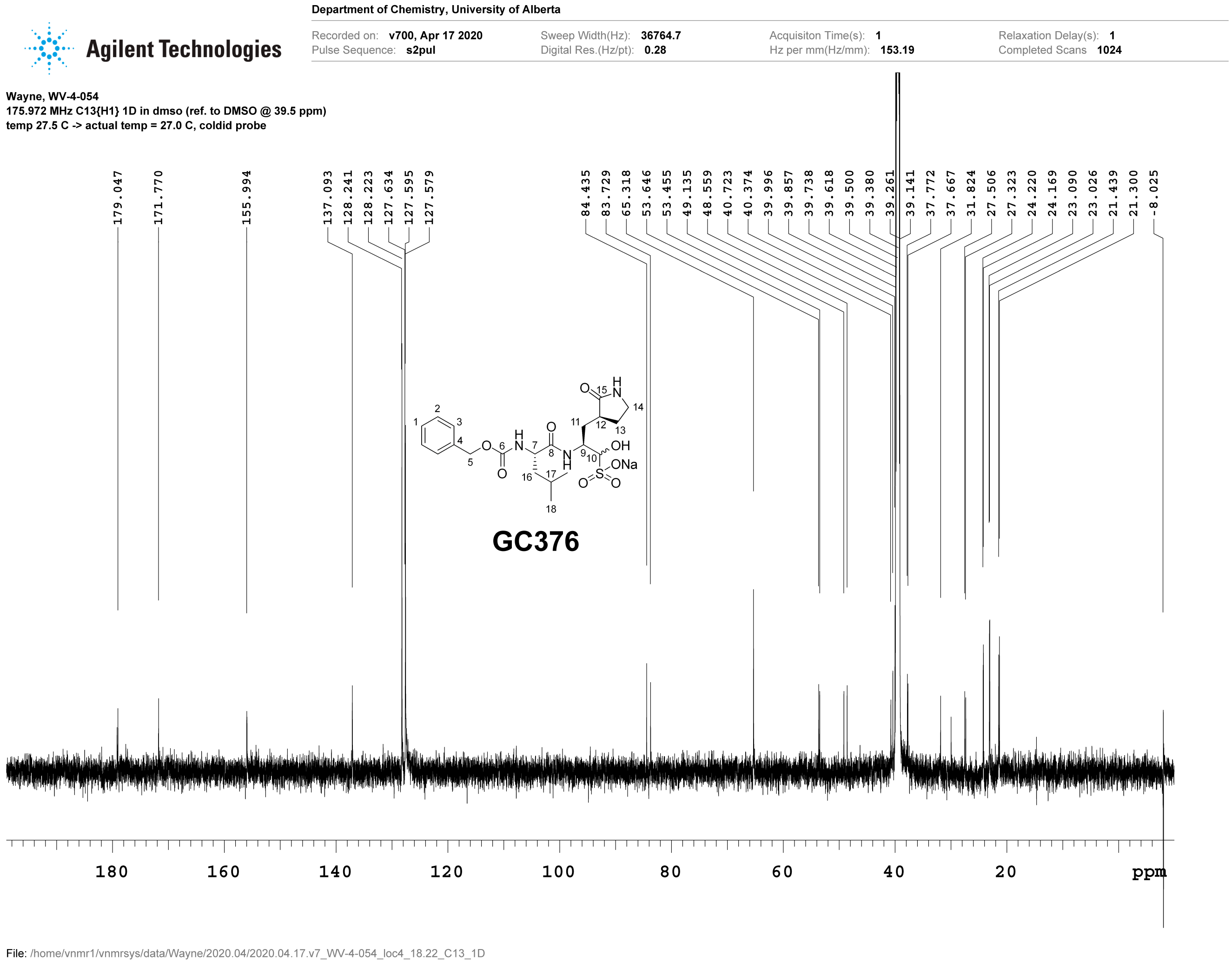

Supplementary Fig. 12. ^{13}C-NMR spectra and molecular structure of GC376. Identity and purity of product resulting from the synthetic steps described in the methods section above can be confirmed. Chemical shift values correspond to values expected of the target compound.

A

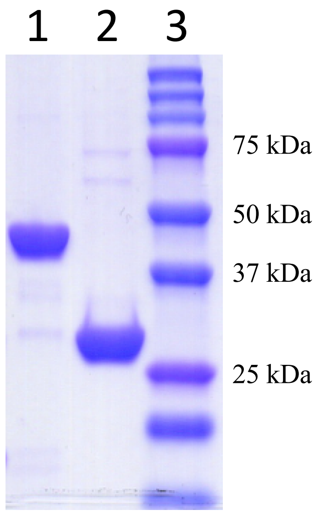

B

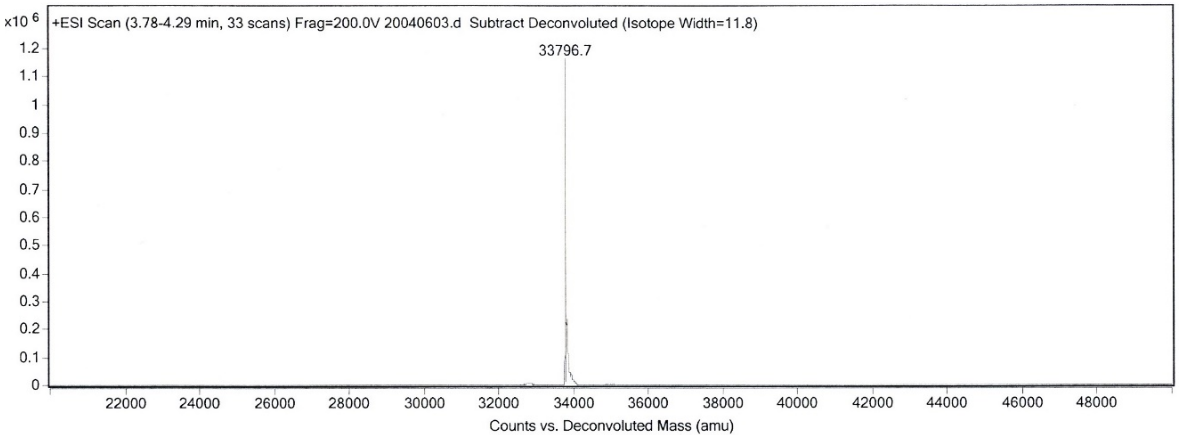

Supplementary Fig. 13. Purification of SARS-CoV-2 M^{pro}. A) Coomassie stained SDS-PAGE of SARS-CoV-2 M^{pro} purification. Lane 1: SUMO-SARS-CoV-2 M^{pro} fusion protein; lane 2: SARS-CoV-2 M^{pro} after cleavage SUMO-tag; lane 3: protein MW ladder. **B)** ESI-MS of purified SARS-CoV-2 M^{pro}.

A

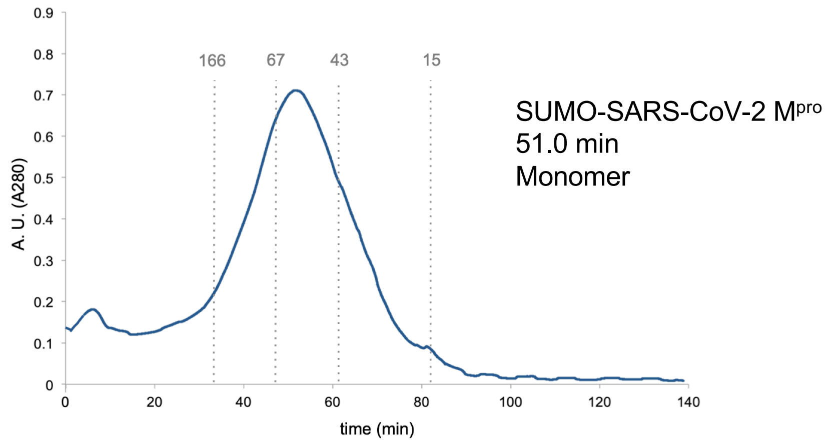

B

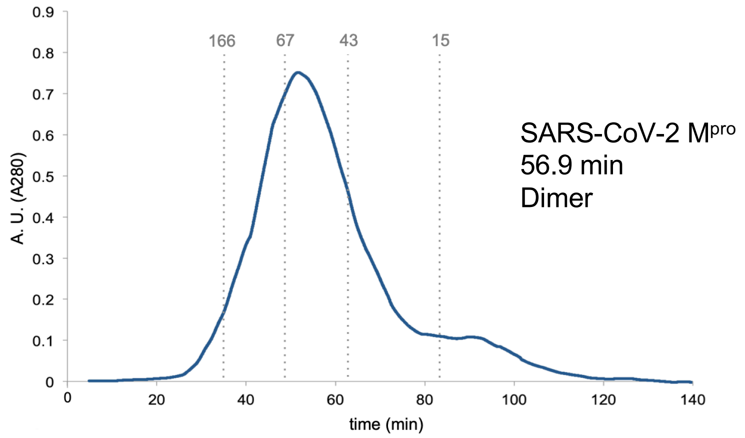

Supplementary Fig. 14. Gel filtration of SUMO-tagged SARS-CoV-2 M^{pro} and SARS-CoV-2 M^{pro}. A) The SUMO-tagged variant eluted as a monomeric species. **B)** The respective free SARS-CoV-2 M^{pro} exists predominantly as dimer (90% dimer). Retention times are provided at peaks. Calibration standards are indicated by grey lines and numbers represent kDa. AU= Absorbance at 280nm.

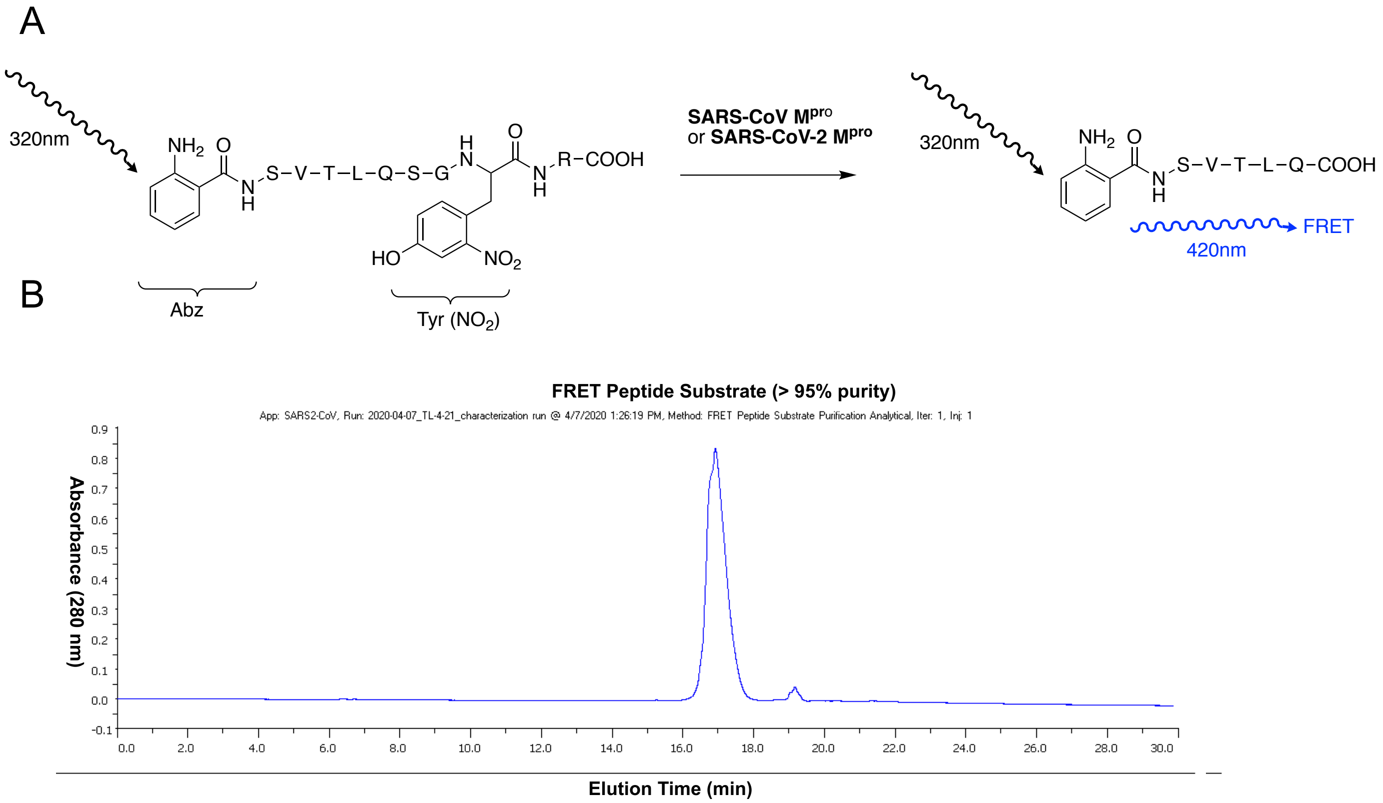

Supplementary Fig. 15. A) Molecular mechanism of the FRET assay involves a peptide 9-mer with fluorophore (Abz) and quencher (Tyr(NO_2)). Upon cleavage by M^{pro} FRET emission at 420nm can be observed. **B)** HPLC chromatogram of FRET-substrate, Abz-SVTLQSG-Y(NO2)-R, indicating a purity >95%.

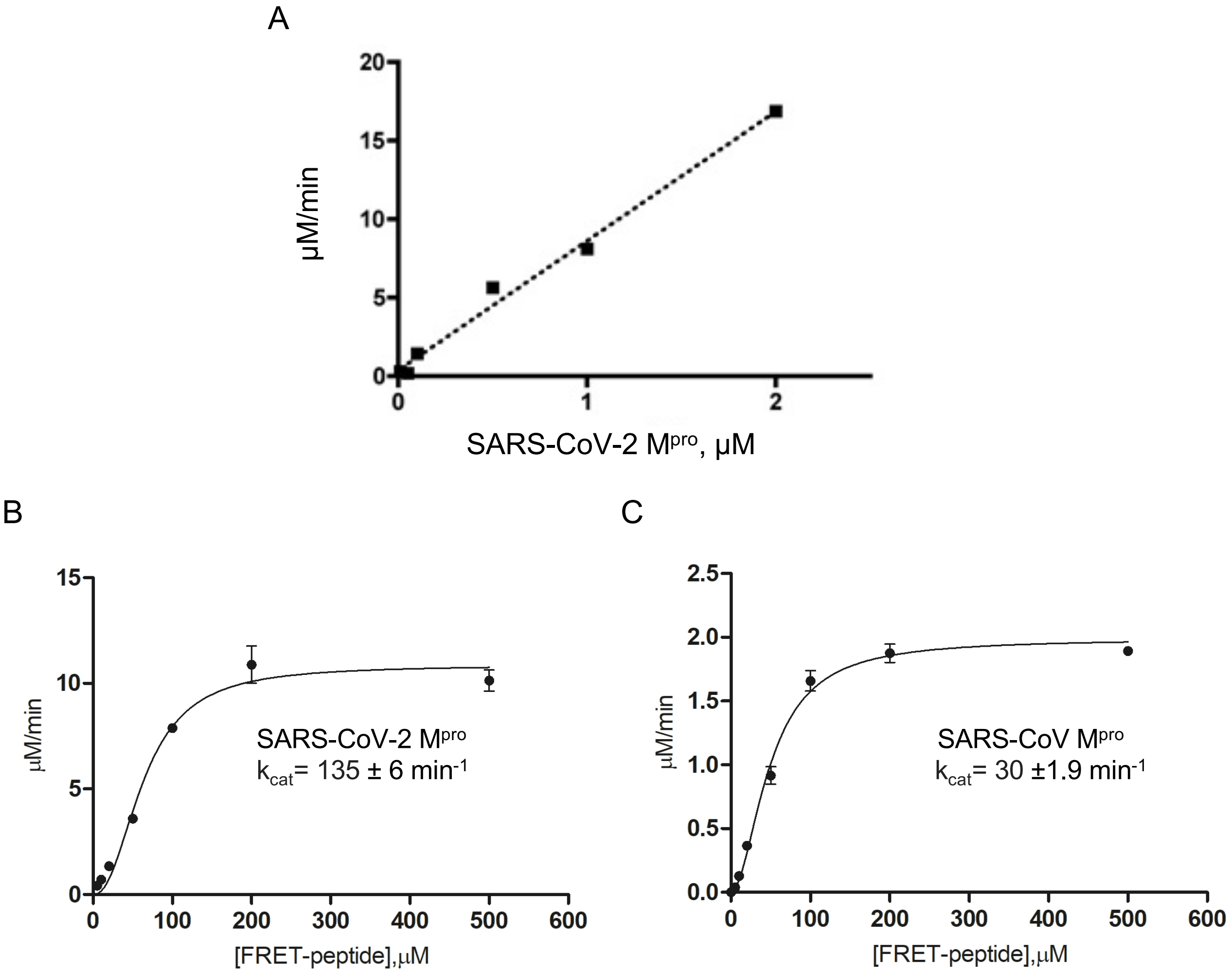

Supplementary Fig. 16. Kinetic parameters of SARS-CoV-2-M^{pro} and SARS M^{pro} . A) Linearity plot of enzyme activity. **B)** Representative Hill plots for Abz-SVTLQSG-Y(NO2)-R cleavage by SARS-CoV-2-M^{pro} and **C)** SARS M^{pro}. Data is represented as mean±SEM of three independent experiments (n=3).

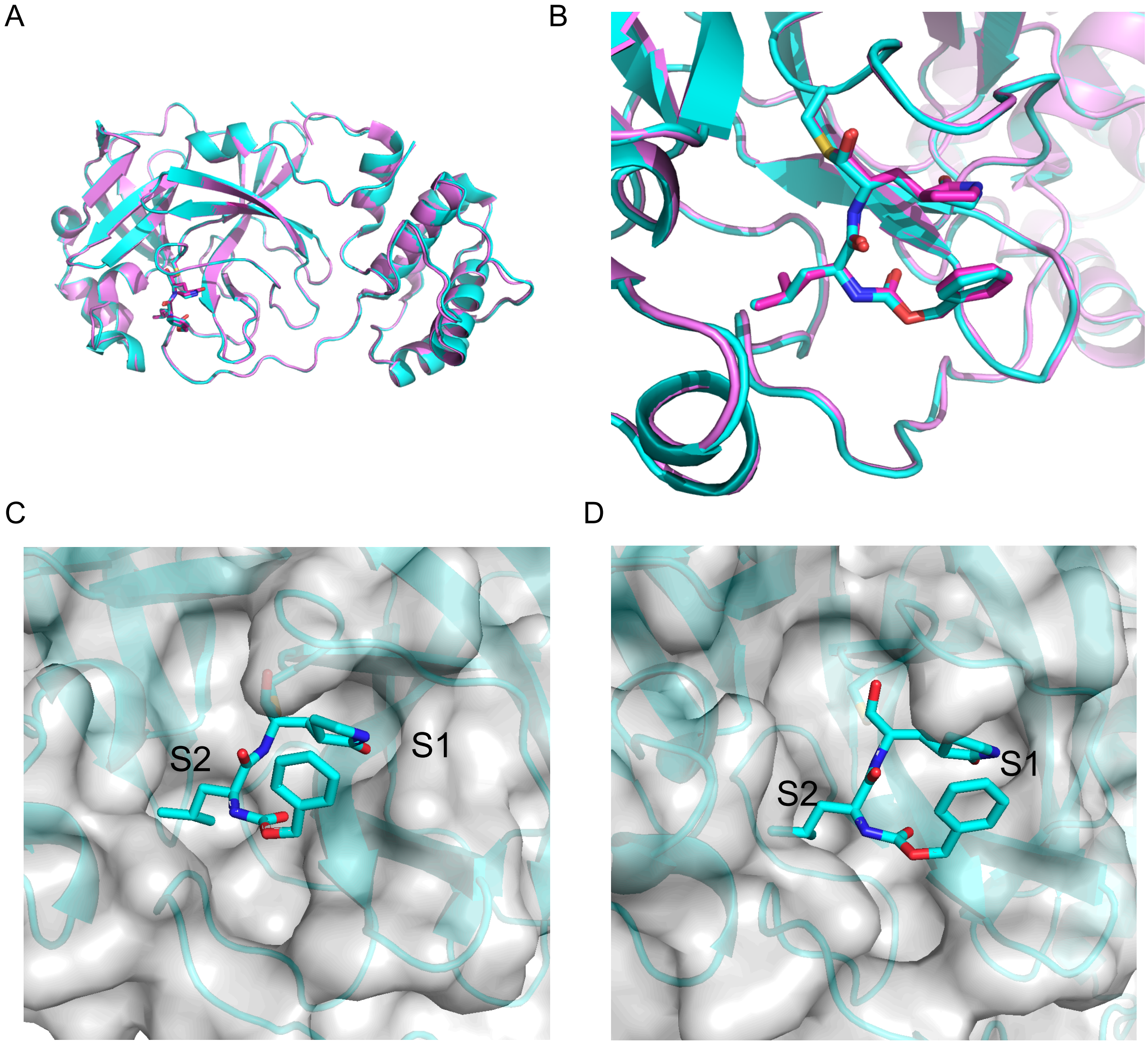

Supplementary Fig. 17. Comparative crystal structures of SARS-CoV-2 M^{pro} with drug GC373 or prodrug GC376. A) Alignment of crystal structure of SARS-CoV-2 M^{pro} soaked with GC373 (6WTK.pdb) or GC376 (6WTJ.pdb). The GC373-derived structure is shown in pink, and the GC376-derived in blue. RMSD 0.28 Å^2 **B)** Active site of both structures (drug and prodrug) show identical inhibitor moiety, demonstrating the bisulphite adducts leave the GC376 compound to form GC373. **C)** and **D)** An examination of the fit in the enzyme subsites (S1 and S2) reveal regions to explore for novel inhibitor development.

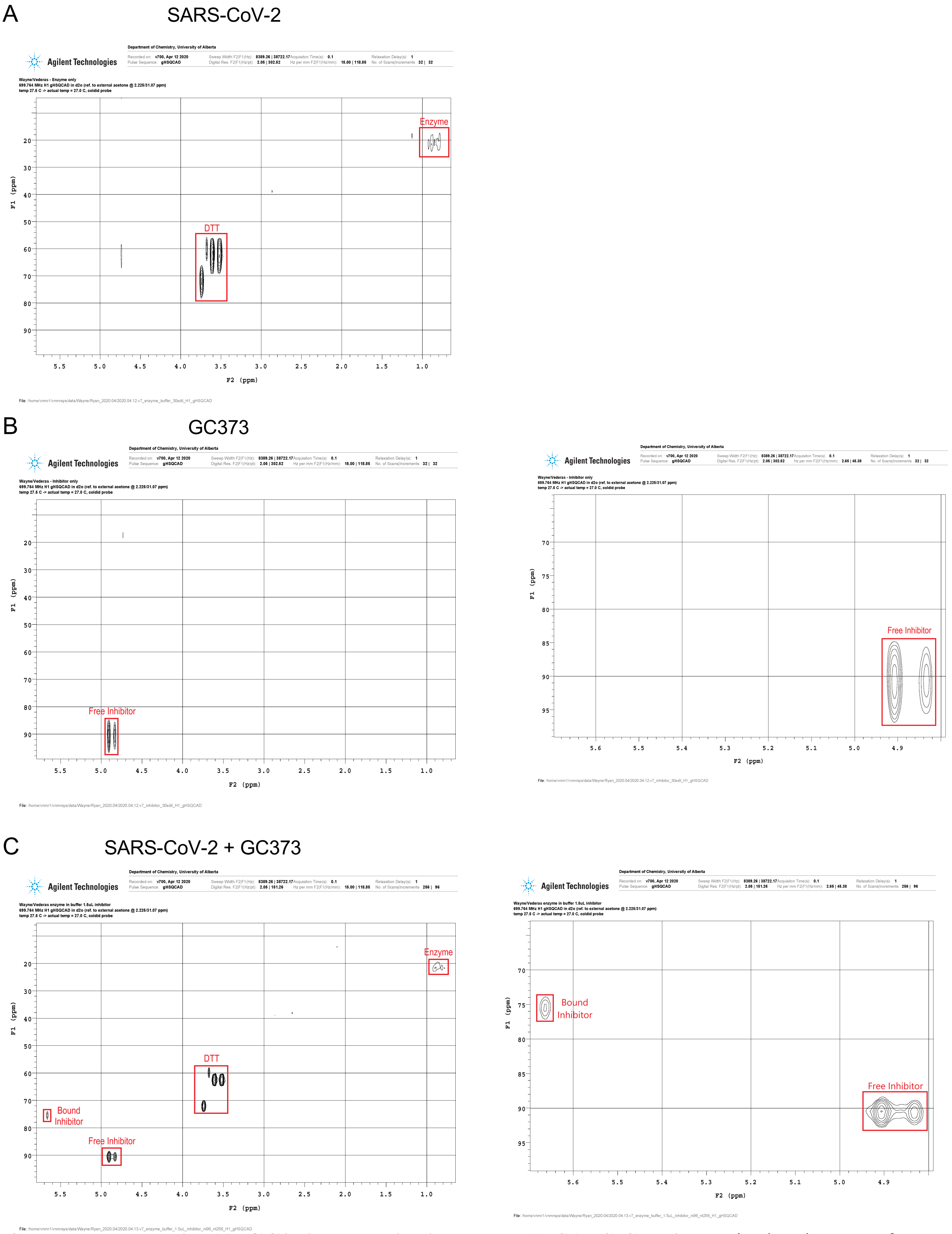

Supplementary Fig. 18. GC373 NMR binding assay. A) SARS-CoV-2 M^{pro} in the absence of GC373 inhibitor. Zoomed in spectra are shown on the right. **B)** GC373 inhibitor in the absence of SARS-CoV-2 M^{pro}. **C)** Co-incubation of SARS-CoV-2 M^{pro} with GC373 inhibitor. A new crosspeak corresponding to the bound inhibitor can be observed, consistent with a hemithioacetal functionality.

	SARS-CoV-2 M^{pro}	SARS-CoV2 M^{pro}-GC373	SARS-CoV2 M^{pro}-GC376
PDB entry	6WTM	6WTK	6WTJ
**Data collection**			
Space group	$P2_1$	C2	C2
Cell dimensions			
a, b, c (Å)	44.84, 53.60, 114.87	113.35, 53.03, 45.23	114.97, 53.81, 45.50
α, β, γ (°)	90, 101.22, 90	90, 102.03, 90	90, 101.74, 90
Resolution (Å)	38.84 - 1.85 (1.91-1.85)	34.04 - 2.00 (2.07-2.00)	39.01 - 1.90 (1.96 - 1.90)
Observations	303947 (28829)	115225 (10007)	141452 (13399)
R_{merge}	0.133 (0.254)	0.146 (0.264)	0.105 (0.341)
$I / \sigma I$	9.1 (1.4)	8.0 (1.0)	10.1 (1.0)
Completeness (%)	98.1 (98.7)	96.64 (96.90)	98.35 (97.92)
Redundancy	6.7 (6.3)	6.5 (5.8)	6.7 (6.5)
CC1/2	99.8 (54.5)	99.60 (29.60)	99.80 (41.50)
**Refinement**			
Resolution (Å)	37.56 - 1.85	31.52-2.00	34.36-1.90
No. reflections	45129	17307	21256
R_{work} / R_{free}	20.52/24.65	20.23/25.25	20.01/23.63
No. atoms	5041	2434	2473
Protein	4761	2367	2386
Ligand/ion	N/A	29	29
Water	280	38	58
B-factors	41.23	61.31	57.70
Protein	40.96	61.43	57.80
Ligand/ion	N/A	60.49	57.09
Water	45.95	54.25	53.94
R.m.s. deviations			
Bond lengths (Å)	0.008	0.009	0.009
Bond angles (°)	1.040	1.40	1.20

*Values in parentheses are for highest-resolution shell. Each data were collected from single crystal

Supplementary Table 1. Data collection and refinement statistics from molecular replacement.

Compound	GC373			GC376		
Main Protease	IC_{50} (µM)	EC_{50} (µM)	CC_{50} (µM)	IC_{50} (µM)	EC_{50} (µM)	CC_{50} (µM)
SARS-CoV M^{pro}	0.07 ±0.02 *		>500 [1]	0.05 ±0.01 *		>500 [1]
SARS-CoV2 M^{pro}	0.40 ±0.05 *	1.5 ±0.3*	>200 *	0.19 ±0.04 *	0.9 ±0.2 *	>200 *
MERS M^{pro}				1.56 ±0.09 [3]	0.9 [4]	
FCoV M^{pro}		0.02 ±0.01[2]	>150 [2]		0.04 ±0.04 [2]	>150 [2]
FCV M^{pro}		>5 [2]	>150 [2]		>5 [2]	>150 [2]
FIPV M^{pro}		0.3±0.1[1]	>500 [1]	0.72 ±0.07 [3]	0.2 ±0.1[1]	

Supplementary Table 2. A comparison of inhibitory concentrations using purified protein in FRET-based assays (IC_{50}), Effective concentration in cellular studies (EC_{50}), and cytotoxicity concentration (CC_{50}) parameters for the M^{pro} of SARS-CoV-2 and other related viruses. FCoV: feline coronavirus; FCV: feline calicivirus; FIPV: Feline infectious peritonitis virus. *: data from this manuscript.

1. Kim Y*, et al.* Broad-spectrum antivirals against 3C or 3C-like proteases of picornaviruses, noroviruses, and coronaviruses. *Journal of virology* 86, 11754-11762 (2012).
2. Kim Y*, et al.* Broad-spectrum inhibitors against 3C-like proteases of feline coronaviruses and feline caliciviruses. *Journal of virology* 89, 4942-4950 (2015).
3. Kim Y*, et al.* Reversal of the Progression of Fatal Coronavirus Infection in Cats by a Broad-Spectrum Coronavirus Protease Inhibitor. *PLoS pathogens* 12, e1005531 (2016).
4. Galasiti Kankanamalage AC*, et al.* Structure-guided design of potent and permeable inhibitors of MERS coronavirus 3CL protease that utilize a piperidine moiety as a novel design element. *European journal of medicinal chemistry* 150, 334-346 (2018).
