# Supplementary material for: Feline coronavirus drug inhibits the main protease of SARS-CoV-2 and blocks virus replication
Source: Nat Commun. 2020 Aug 27;11:4282. doi: 10.1038/s41467-020-18096-2 (PMC7453019; doi:10.1038/s41467-020-18096-2)
Supplement: Supplementary file 2 — Reporting Summary [file 41467_2020_18096_MOESM2_ESM.pdf]

nature research

Corresponding author(s): M. Joanne Lemieux, John C. Vederas, D. Lorne Tyrrell

Last updated by author(s): May 3, 2020

Reporting Summary

Nature Research wishes to improve the reproducibility of the work that we publish. This form provides structure for consistency and transparency in reporting. For further information on Nature Research policies, see Authors & Referees and the Editorial Policy Checklist .

Statistics

For all statistical analyses, confirm that the following items are present in the figure legend, table legend, main text, or Methods section.

n/a	Confirmed	
[ ]	[x]	The exact sample size (*n*) for each experimental group/condition, given as a discrete number and unit of measurement
[ ]	[x]	A statement on whether measurements were taken from distinct samples or whether the same sample was measured repeatedly
[x]	[ ]	The statistical test(s) used AND whether they are one- or two-sided<br>*Only common tests should be described solely by name; describe more complex techniques in the Methods section.*
[x]	[ ]	A description of all covariates tested
[x]	[ ]	A description of any assumptions or corrections, such as tests of normality and adjustment for multiple comparisons
[ ]	[x]	A full description of the statistical parameters including central tendency (e.g. means) or other basic estimates (e.g. regression coefficient) AND variation (e.g. standard deviation) or associated estimates of uncertainty (e.g. confidence intervals)
[x]	[ ]	For null hypothesis testing, the test statistic (e.g. *F*, *t*, *r*) with confidence intervals, effect sizes, degrees of freedom and *P* value noted<br>*Give P values as exact values whenever suitable.*
[x]	[ ]	For Bayesian analysis, information on the choice of priors and Markov chain Monte Carlo settings
[x]	[ ]	For hierarchical and complex designs, identification of the appropriate level for tests and full reporting of outcomes
[x]	[ ]	Estimates of effect sizes (e.g. Cohen's *d*, Pearson's *r*), indicating how they were calculated

Our web collection on statistics for biologists contains articles on many of the points above.

Software and code

Policy information about availability of computer code

Data collection	Blu-Ice BL17U1, WebIce
Data analysis	XDS (BUILT=20200417); CCP4 (v7.0.077); Coot (v0.8.6.1); Phenix (v1.18.1-3855); PyMOL (v1.7.6.5); GraphPad Prism (v8.3.1); Microsoft Excel (v16.35); PyMOL (v2.3.4); Agilent Mass Hunter Qualitative Analysis software Package (version B.03.01 SP3)

For manuscripts utilizing custom algorithms or software that are central to the research but not yet described in published literature, software must be made available to editors/reviewers. We strongly encourage code deposition in a community repository (e.g. GitHub). See the Nature Research guidelines for submitting code & software for further information.

Data

Policy information about availability of data

All manuscripts must include a data availability statement. This statement should provide the following information, where applicable:
- Accession codes, unique identifiers, or web links for publicly available datasets
- A list of figures that have associated raw data
- A description of any restrictions on data availability

The datasets generated during the current study are available at https://www.rcsb.org; NMR data can be accessed here: http://deposit.bmrb.wisc.edu/author_view/BMRB/50262_hy_lssvgcxu.str].

Field-specific reporting

Please select the one below that is the best fit for your research. If you are not sure, read the appropriate sections before making your selection.

[x] Life sciences [ ] Behavioural & social sciences [ ] Ecological, evolutionary & environmental sciences

October 2018

For a reference copy of the document with all sections, see nature.com/documents/nr-reporting-summary-flat.pdf

Life sciences study design

All studies must disclose on these points even when the disclosure is negative.

Sample size	For kinetic analysis and cell studies sample size was chosen with n of three or greater, and standard error or standard deviation was calculated using GraphPad (v.8.3.1)
Data exclusions	No data points were excluded from kinetic analysis, IC50 determination, or cell based assays.
Replication	The experiments for all assays were repeated as independent experiments at least 3 times. All replicates were sucessfull. The reproducibility is reflected in error bars.<br>Kinetic analysis (table 1 and Fig. S5) were performed as with three biological replicates and these data were used to calculate mean values and standard errors.<br>IC50 measurements (Fig. 1B, C) were carried out with three biological replicates for each data point to calculate the mean and SE .<br>EC50 assay (Fig. 4A and B) were carried out with three independent biological replicates in 2 separate experiments. Error bars represent SD.<br>Cytotoxicity assays (Fig.4B and B) were done with four independent biological replicates in three separate experiments. Error bars represent SD.<br>The antiviral activity assay (Figure 4C and D) was carried out with three independent biological replicates. Error bars represent SD.
Randomization	Randomization does not apply to the current study, as these are kinetics and observation studies.
Blinding	Blinding does not apply to the current study, as these are kinetics and observation studies.

Reporting for specific materials, systems and methods

We require information from authors about some types of materials, experimental systems and methods used in many studies. Here, indicate whether each material, system or method listed is relevant to your study. If you are not sure if a list item applies to your research, read the appropriate section before selecting a response.

Materials & experimental systems

n/a	Involved in the study	
☒	☐	Antibodies
☐	☒	Eukaryotic cell lines
☒	☐	Palaeontology
☒	☐	Animals and other organisms
☒	☐	Human research participants
☒	☐	Clinical data

Methods

n/a	Involved in the study	
☒	☐	ChIP-seq
☒	☐	Flow cytometry
☒	☐	MRI-based neuroimaging

Eukaryotic cell lines

Policy information about cell lines

Cell line source(s)	African green monkey origin, Vero from ATCC; A549, human male adenocarcinoma of alveolar basal epithelial cells from ATCC
Authentication	All cells were from ATCC with authentication. The authentication was performed by morphology check under microscopes and growth curve analysis.
Mycoplasma contamination	We confirm that all cells were tested as mycoplasma negative.
Commonly misidentified lines<br>(See ICLAC register)	No commonly misidentified cell lines were used.
